# Optimization of Molecular Methods for Detecting Duckweed-Associated Bacteria

**DOI:** 10.3390/plants12040872

**Published:** 2023-02-15

**Authors:** Kenneth Acosta, Shawn Sorrels, William Chrisler, Weijuan Huang, Sarah Gilbert, Thomas Brinkman, Todd P. Michael, Sarah L. Lebeis, Eric Lam

**Affiliations:** 1 Department of Plant Biology, Rutgers the State University of New Jersey, New Brunswick, NJ 08901, USA; 2 Environmental Molecular Sciences Laboratory (EMSL), Pacific Northwest National Laboratory (PNNL), Richland, WA 99354, USA; 3 Institute of Nanfan & Seed Industry, Guangdong Academy of Sciences, Guangzhou 510316, China; 4 The Plant Molecular and Cellular Biology Laboratory, The Salk Institute for Biological Studies, La Jolla, CA 92037, USA; 5 Department of Plant, Soil and Microbial Sciences, Michigan State University, East Lansing, MI 48824, USA; 6 Department of Microbiology and Molecular Genetics, Michigan State University, East Lansing, MI 48824, USA; 7 Plant Resilience Institute, Michigan State University, East Lansing, MI 48824, USA

**Keywords:** plant-microbe interactions, plant-bacteria associations, bacterial colonization, duckweed, duckweed-associated bacteria, RISA, *Azospirillum brasilense* Sp7, *Azospirillum brasilense* Sp245, strain-specific primers, bead-beating

## Abstract

The bacterial colonization dynamics of plants can differ between phylogenetically similar bacterial strains and in the context of complex bacterial communities. Quantitative methods that can resolve closely related bacteria within complex communities can lead to a better understanding of plant–microbe interactions. However, current methods often lack the specificity to differentiate phylogenetically similar bacterial strains. In this study, we describe molecular strategies to study duckweed–associated bacteria. We first systematically optimized a bead-beating protocol to co-isolate nucleic acids simultaneously from duckweed and bacteria. We then developed a generic fingerprinting assay to detect bacteria present in duckweed samples. To detect specific duckweed–bacterium associations, we developed a genomics-based computational pipeline to generate bacterial strain-specific primers. These strain-specific primers differentiated bacterial strains from the same genus and enabled the detection of specific duckweed–bacterium associations present in a community context. Moreover, we used these strain-specific primers to quantify the bacterial colonization of duckweed by normalization to a plant reference gene and revealed differences in colonization levels between strains from the same genus. Lastly, confocal microscopy of inoculated duckweed further supported our PCR results and showed bacterial colonization of the duckweed root–frond interface and root interior. The molecular methods introduced in this work should enable the tracking and quantification of specific plant-microbe associations within plant-microbial communities.

## 1. Introduction

Lemnaceae, commonly known as duckweed, is a family of freshwater aquatic plants [1]. Their small size, short doubling time, growth habitat, and reduced morphology put forth duckweed as a model system to study plant–microbe interactions. Indeed, many similarities can be found between the structuring of duckweed-associated bacterial (DAB) communities and terrestrial plant bacterial communities. For example, both terrestrial plants and duckweed host distinct bacterial communities when compared to the host environment, demonstrating that selection strongly shapes the bacterial communities of both terrestrial plants and duckweed [2,3,4,5]. Moreover, similar bacterial taxa are found among terrestrial plant bacterial communities and DAB communities, suggesting bacterial adaptation to these plant habitats [3]. Therefore, studying duckweed–bacteria interactions may help reveal conserved mechanisms involved in plant–bacteria interactions.

The study of plant–bacteria interactions is complicated by many factors. One factor is the functional diversity found among phylogenetically similar bacteria associated with plants. Despite their similar phylogeny, these related bacteria can interact differently with plant hosts and may serve diverse roles in plant microbial communities. Community surveys of plant bacterial communities show that bacteria of the same genus can have different colonization dynamics in different plant tissues and developmental stages [6]. Other community surveys show bacteria of the same family can have distinct plant host preferences [7]. In support of these community surveys, functional studies show bacterial strains from the same genus can colonize plants at different concentrations and protect against disease to different degrees [8,9]. Another factor that adds complexity to plant–microbe interactions is the presence of microbe–microbe interactions within microbial communities [10]. Bacteria may readily colonize plants when no other microbes are present, but could fail to stably colonize plants in a community context [11]. The presence of microbe–microbe interactions in microbial communities is a major reason why many bacteria display plant-growth-promoting behavior in the laboratory in mono-association studies but not in field trials when natural microbial communities are present [12]. Thus, differentiating phylogenetically similar bacteria in diverse contexts will be important to unravel the complexity of plant–bacteria interactions.

Different methods can be used to detect plant-bacteria associations, including culture-dependent methods, microscopy, and molecular approaches [13,14]. Together, these methods differ in the information they provide and in the context in which they can be applied. The colony-forming units (CFU) assay is a culture-dependent method used to quantify live bacteria. In the context of plant-bacteria interactions, this method has typically been used to quantify the colonization of plants by single bacterial isolates [15,16,17,18], but with the implementation of selective culture media, members in a small community of plants and bacteria can also be distinguished [19]. Still, this method can be laborious, imprecise, and cannot be used to quantify bacteria found in complex microbial communities [14]. In contrast to the CFU assay, microscopy is a qualitative approach used to observe the spatial and temporal colonization dynamics of bacteria on plants [20]. Its application has revealed the presence of colonization hotspots on plants and different colonization patterns between bacteria when applied individually to plant tissues [21,22,23,24]. However, microscopy techniques commonly use generic fluorescent stains or oligonucleotide probes that cannot detect specific interactions within a bacterial community [13,25,26]. An alternative microscopy approach involves labeling and monitoring a bacterial strain of interest with an in vivo reporter gene, such as GFP or GUS, but this application can also be laborious and is dependent on the transformability of the bacterium of interest [27,28,29]. Thus, the CFU assay and microscopy methods are often used to study bipartite plant–bacterium interactions, since they lack the specificity required to study the interactions between plants and specific members in complex bacterial communities.

The most common method to detect bacteria in complex communities is 16S rDNA amplicon sequencing, in which variable regions of the 16S rRNA gene are selectively amplified and commonly sequenced by short-read sequencing technologies [30,31,32]. Initially, 16S rDNA amplicon sequencing provided the relative abundance of bacteria within communities, but recent innovations allow for the absolute abundance of community members to be quantified [33,34,35]. Other recent innovations include the application of long-read sequencing technologies to sequence full-length 16S rRNA gene sequences, since full-length sequences demonstrate higher resolution than partial sequences [36,37,38]. Initially, the high error rate of long-read sequencing technologies limited their use in 16S rDNA amplicon sequencing, but this error rate has diminished with the implementation of circular consensus sequencing and denoising algorithms tailored to long-read technologies [37,39]. Despite these innovations, this molecular approach is still limited by the extent of polymorphisms in the 16S rRNA gene, which can distinguish between bacterial families and genera but often lacks resolution between strains of the same bacterial species and between some closely related bacterial species [40,41,42]. This is also exemplified in community surveys using full-length 16S rRNA gene sequences, where some reads cannot be assigned at the species level and some closely related bacterial species cannot be resolved [37,38]. In addition to low resolution, some bacterial taxa contain multiple non-identical copies of the 16S rRNA gene, further complicating the differentiation of closely related bacteria with this molecular approach [43,44]. For these reasons, a different approach, not reliant on the sequencing of the 16S rRNA gene, is needed to differentiate phylogenetically similar bacteria in community scenarios.

One molecular approach that can track and quantify specific bacterial isolates of interest within complex communities is real-time PCR (qPCR) [13,14,45]. In this context, strain-specific primers are designed for qPCR using unique sequences from the bacterium of interest. Initially, sequences from the 16S rRNA gene or sequence-characterized amplified regions were used to design strain-specific primers [46,47], but with current technologies entire bacterial genomes can be examined for strain-specific sequences [48,49,50]. As a result, the qPCR approach can provide high specificity and sensitivity, demonstrated by its ability to detect specific bacterial strains in non-sterile soils [51,52,53]. However, results from this molecular method are dependent on the efficiency of the method used to extract DNA and the resulting sample quality. Furthermore, reported strategies to design primers either involve custom protocols [51,53] or fragmenting bacterial genomes into smaller sequences to search against public databases [48,49]. These approaches can be inefficient if strain-specific sequences are desired for many bacteria. Therefore, a broadly applicable and efficient strategy for designing strain-specific primers is preferred to fully realize the potential of qPCR for studying specific plant–bacterium associations.

In this study, we optimized and applied molecular methods to detect duckweed-associated bacteria in simple (i.e., binary) or community contexts. To apply these molecular methods, we first systematically optimized a bead-beating protocol to co-isolate nucleic acids simultaneously from both duckweed and bacteria. Second, we combined ribosomal intergenic spacer analysis (RISA) and PCR of a plant-specific marker to detect bacteria associated with duckweed. Third, we used publicly available computational tools and developed an accessible computational pipeline to design strain-specific primers for different bacteria. These primers were able to detect and quantify the associations of duckweed with the respective bacteria, either alone or in the presence of ambient microbial communities from wastewater samples. Lastly, we used confocal microscopy as a complementary approach to describe bacterial colonization dynamics on the duckweed *Lemna minor*. The molecular methods introduced in this work could enable high-resolution, quantitative studies of duckweed–associated bacteria in diverse contexts.

## 2. Results

### 2.1. Selection of Duckweed Strain and Bacterial Isolates

Duckweed and bacteria were obtained to study the colonization dynamics of bacteria on duckweed. The duckweed strain *Lemna minor* 5576 (Lm5576) was acquired from the Rutgers Duckweed Stock Cooperative (RDSC; New Brunswick, NJ, USA). This duckweed strain has been previously used to study duckweed-associated bacterial communities [3]. Bacteria originating from different hosts were acquired (File S1). One of the duckweed-associated bacterial (DAB) isolates, *Microbacterium* sp. RU370.1 (DAB 1A), was isolated from Lm5576 and can produce the phytohormone indole-3-acetic acid (IAA) as well as colonize and affect the root development of *Arabidopsis thaliana* [54,55]. Another bacterial isolate was retrieved from the seaweed *Ulva fasciata*. This seaweed-associated bacterium, *Bacillus simplex* RUG2-6 (G2-6), was hypothesized to be a weak colonizer of duckweed due to the large evolutionary divergence between macroalgae and angiosperms. Two bacterial isolates of well-characterized plant colonizers, an epiphyte *Azospirillum brasilense* Sp7 (Sp7) and a known endophyte *Azospirillum baldaniorum* Sp245 (Sp245), were acquired to act as positive colonization controls [56]. Both these strains (Sp7 and Sp245) contain similar 16S rRNA gene sequences (97–99.9% identity) and were initially classified as *Azospirillum brasilense*, but recent phylogenomic analyses show Sp245 belongs to the novel species named *Azospirillum baldaniorum* [57]. Together, these bacteria were used to inoculate duckweed to study their colonization dynamics of axenic Lm5576.

### 2.2. Systematic Optimization of a Nucleic Acid Extraction Method for Duckweed and Bacteria

To characterize bacterial colonization of duckweed using molecular methods, an optimized protocol for efficiently isolating nucleic acid simultaneously from duckweed and different bacteria was developed. First, nucleic acid extraction was compared between bead-beating and homogenization with a mortar and pestle using a modified CTAB protocol [58] (Figure S1). While the mortar and pestle extracted more nucleic acids from Lm5576 than bead-beating, only bead-beating was able to extract both duckweed and bacterial nucleic acids. Therefore, bead-beating was selected as the physical lysis method for nucleic acid extraction. Various parameters of the bead-beating protocol were then modified to improve duckweed and bacteria nucleic acid extraction. First, three different sizes of beads were compared for their ability to extract plant and bacterial nucleic acids (Figures S2 and S3). These tests showed 1.7 mm zirconium beads were the most effective at homogenizing duckweed tissues and extracting duckweed nucleic acids, and 100 µm silica beads were the best for extracting bacterial nucleic acids. Furthermore, a combination of different-sized beads effectively extracted nucleic acids from both duckweed and bacteria. Therefore, a combination of different-sized beads was used for the bead-beating protocol. Chloroform and a heating step at 65 °C were then added to the lysis step to test their ability to improve nucleic acid extraction (Figure S4A). Both chloroform and heating improved nucleic acid extraction from duckweed. Bead-beating was also performed at different temperatures with and without the addition of a reducing agent to test for improvement of nucleic acid extraction (Figure S4B,C). All conditions resulted in high yields of intact nucleic acids, but bead-beating at room temperature without a reducing agent showed a slightly higher yield of nucleic acids and with DNA higher in apparent molecular weight. Lastly, nucleic acid extractions were performed using various incubation times in the lysis buffer with different bacteria, including isolates from monoderm (Firmicutes and Actinobacteria) and diderm (Proteobacteria) bacterial phyla (Figure S5). Nucleic acids were extracted from both monoderm and diderm bacteria, and the nucleic acid yield increased with longer incubation times in the lysis buffer for some isolates. From these experiments, an optimized bead-beating protocol was developed that could be used to effectively extract nucleic acids from duckweed inoculated with diverse bacteria types.

### 2.3. Establishment of a PCR-Based DNA Fingerprinting Assay for Detecting Duckweed–Associated Bacteria

rRNA intergenic spacer analysis (RISA) is a PCR-based method that amplifies the intergenic spacer region between 16S and 23S rRNA genes. This region can vary in copy number, sequence, and length among bacterial species. As a result, RISA can be used to estimate bacterial community composition by generating community fingerprints [59] and for rapid, universal bacterial typing [60]. In this study, RISA was applied as a simple molecular approach to detect duckweed-associated bacteria. Different RISA primer sets were tested for their ability to amplify DNA from different bacterial species and duckweed (File S2, Figure S6). 16S-e1390f and 23S-e130r primers produced distinct fingerprints between bacterial species while importantly they did not show significant amplification products from Lm5576 DNA under our conditions. Therefore, these primers were selected for detecting bacterial colonization of Lm5576. In addition to RISA, a plant-specific marker was used to compare the relative amount of Lm5576 genomic DNA between samples and to control for sample quality. Primers were designed for detecting the single-copy *Lemna minor* ortholog of the plant-specific *LEAFY* gene (*LmLFY*), which is a master transcription factor for flowering control (File S3). PCR using *LmLFY* primers specifically detected and allowed visual estimation of the relative quantity of Lm5576 DNA between samples (Figure S7). Both RISA PCR and *LmLFY* PCR were used in concert to monitor bacterial colonization of Lm5576. This strategy is subsequently referred to as “attachment PCR”.

### 2.4. Standardization and Validation of Attachment PCR Assay for Detecting Duckweed–Associated Bacteria

**Figure 1:**
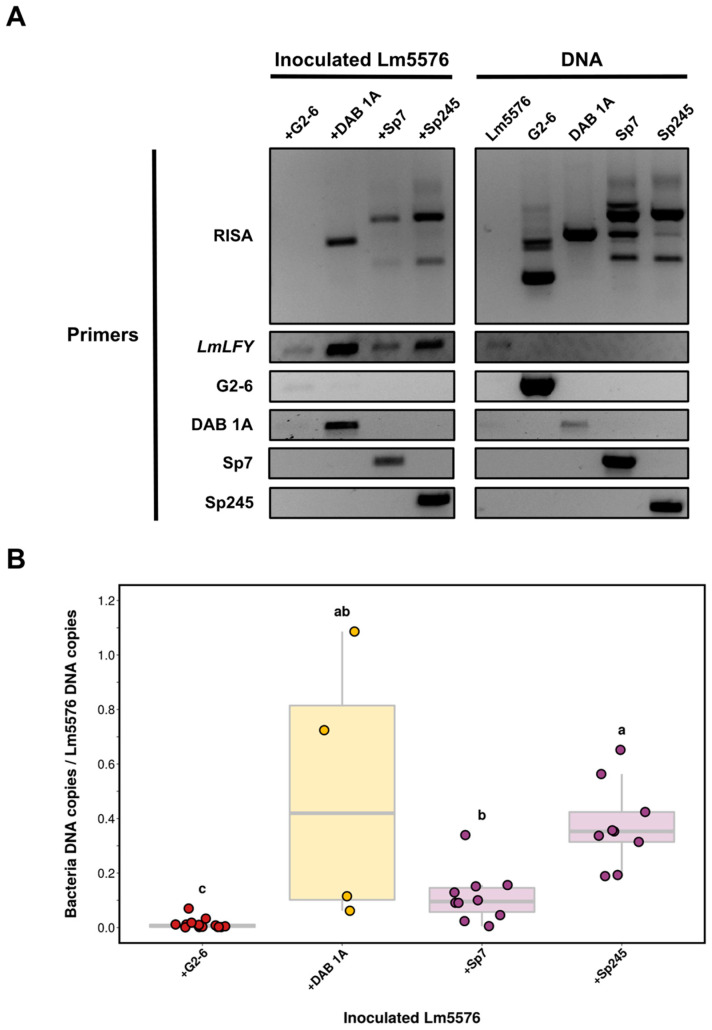
Molecular detection of duckweed-associated bacteria. Axenic Lm5576 was inoculated with different bacteria in 0.5X SH media. After seven days, inoculated Lm5576 tissue was collected, washed with sterile water, and nucleic acids were isolated for analysis. (**A**) Representative gel electrophoresis results of end-point PCR using RISA, *LmLFY*, and strain-specific primers (File S2). RISA PCR fingerprints from inoculated Lm5576 samples were compared to the respective DNA controls from Lm5576 and bacteria alone. Samples: +G2-6 = Lm5576 inoculated with *Bacillus simplex* RUG2-6, +DAB 1A = Lm5576 inoculated with *Microbacterium* sp. RU370.1, +Sp7 = Lm5576 inoculated with *Azospirillum brasilense* Sp7, +Sp245 = Lm5576 inoculated with *Azospirillum baldaniorum* Sp245. Primers: RISA = PCR using 16S-1390f and 23S-e130r primers, *LmLFY* = PCR using LmLFY-F and LmLFY-R primers specific to Lm5576, DAB 1A = PCR using AmRU370.1-F and AmRU370.1-R primers specific to *Microbacterium* sp. RU370.1, G2-6 = PCR using BsRUG2.6-F and BsRUG2.6-R primers specific to *Bacillus simplex* RUG2-6, Sp7 = PCR using AbSp7-F and AbSp7-R primers specific to *Azospirillum brasilense* Sp7, Sp245 = PCR using AbSp245-F and AbSp245-R primers specific to *Azospirillum baldaniorum* Sp245. (**B**) Bacterial association with Lm5576 was quantified using real-time PCR. Bacterial DNA copy number was determined for each inoculated Lm5576 sample and normalized to Lm5576 DNA copy number. Different colors were used for the different bacterial genera. Each data point represents an experimental repeat except for +G2-6, for which each sample was measured twice. A significant difference was found in colonization levels between bacteria (Kruskal–Wallis, *p*-value = 4.58 × 10^−6^). Pairwise comparisons were performed using Dunn’s test, and results are displayed as compact letters (a–c). Bacteria with significantly different colonization levels from each other, according to Dunn’s test (FDR < 0.05), do not share any letters.

Attachment PCR was used to detect and compare the colonization of Lm5576 by G2-6, DAB 1A, Sp7, and Sp245 bacterial strains (Figure 1A). Axenic Lm5576 plants were inoculated with bacteria for seven days. After seven days, inoculated Lm5576 tissue was collected, rinsed with sterile water, and nucleic acids were isolated using the bead-beating protocol described above. Isolated DNA from pure bacterial cultures and sterile Lm5576 were used as controls to compare RISA fingerprints from inoculated Lm5576 samples. RISA PCR did not generate any banding pattern from axenic Lm5576 DNA, whose sterility was verified by culturing on LB agar plates (Section 5.1). RISA PCR of bacterial DNA controls produced distinct fingerprints between G2-6, DAB 1A, and *Azospirillum* strains (Sp7 and Sp245). *LmLFY* PCR of DNA controls only produced a PCR product from the axenic Lm5576 DNA control and none from bacterial DNA controls. All inoculated Lm5576 samples showed *LmLFY* PCR products, indicating good sample quality and the presence of Lm5576 DNA for reference. RISA PCR of Lm5576 inoculated with G2-6 sample did not generate any bacterial fingerprint, suggesting G2-6 was either not able to colonize Lm5576 or colonized Lm5576 at a low concentration not detectable by RISA PCR. RISA PCR of Lm5576 inoculated with DAB 1A produced a fingerprint consisting of a single PCR band that matched the fingerprint of DAB 1A DNA, demonstrating that DAB 1A colonized Lm5576. RISA PCR of Lm5576 inoculated with Sp7 or Sp245 produced a similar fingerprint consisting of two major PCR bands. These two bands were the most prominent PCR bands found in RISA PCR of Sp7 and Sp245 DNA controls indicating Sp7 and Sp245 were both able to colonize Lm5576. The higher concentrations of DNA used for Sp7 and Sp245 DNA controls may explain why the additional PCR bands were not clearly observed in Lm5576 inoculated with Sp7 or Sp245. Overall, attachment PCR showed that DAB 1A, Sp7, and Sp245 could colonize Lm5576 at detectable levels. The exact matching of RISA PCR fingerprints between inoculated Lm5576 samples and DNA controls confirmed the colonization of Lm5576 by the respective bacteria. In addition, this exact matching suggested no contaminating bacteria were present. Therefore, fingerprint matching between RISA PCR of inoculated duckweed and DNA controls can be used to confirm what bacteria are present in duckweed samples.

### 2.5. Computational Pipeline for Primer Design to Detect and Quantify Specific Duckweed-Associated Bacteria

Attachment PCR using RISA and *LmLFY* primer sets detected the colonization of Lm5576 by different bacteria, but it was unable to differentiate strains of the same genus (i.e., Sp7 and Sp245). To distinguish between closely related bacteria, a genomics-enabled approach was taken where strain-specific primers for end-point PCR were designed for each bacterium using available computational pipelines (File S2). For this approach, genomes of G2-6 and DAB 1A were sequenced and the genomes of Sp7 and Sp245 were retrieved from public databases (File S1). The strain-specific primers designed from this pipeline were used for PCR of DNA controls to validate their specificity (Figure 1A). As expected, strain-specific PCR of DNA controls uniquely detected the target bacteria and differentiated Sp7 and Sp245 strains. Strain-specific PCR of inoculated Lm5576 samples showed DAB 1A, Sp7, and Sp245 significantly colonized Lm5576. G2-6-specific PCR showed a faint amplification product in the Lm5576 sample inoculated with G2-6, in contrast to RISA PCR results, suggesting G2-6 may be able to attach to Lm5576 tissues at low concentrations not detectable by RISA PCR. These results show that PCR using bacterial strain-specific primers can distinguish between phylogenetically similar bacterial strains and can be used to detect specific duckweed–bacterium associations.

While end-point PCR with strain-specific primers detected specific duckweed–bacterium associations, it could not be used to accurately quantify bacterial colonization levels. To quantify bacterial colonization of Lm5576, bacterial strain-specific primers were designed for real-time PCR (qPCR) assays using a computational pipeline developed in this study called UniAmp (Section 5.8, Figure S8, File S4). For this computational pipeline, unique genomic sequences were identified and retrieved for each bacterial genome. These unique sequences were then used for optimal primer design. Bacterial strain-specific primers designed by this pipeline were then used to determine bacterial DNA copy number in respective inoculated Lm5576 samples. Bacteria DNA copy number was then normalized to the Lm5576 DNA copy number of the inoculated Lm5576 sample using Lm5576-specific primers. This approach is referred to as “attachment qPCR”. Attachment qPCR showed a significant difference (Kruskal–Wallis, *p*-value = 4.58 × 10^−6^) in the colonization of Lm5576 between the bacteria tested (Figure 1B). Attachment qPCR showed G2-6 associated with Lm5576 in significantly lower concentrations compared to the other bacteria tested (Dunn’s test, *p*-value < 0.005 for all comparisons), similarly to what was found qualitatively by end-point strain-specific PCR (Figure 1A). DAB 1A and Sp245 had the highest colonization levels on Lm5576. However, DAB 1A displayed higher variability between experimental repeat samples, so no significant difference was established compared to Sp7 or Sp245. Sp7 colonized Lm5576 at significantly lower concentrations than Sp245 (Dunn’s test, *p*-value < 0.05). In conclusion, attachment qPCR revealed a significant difference in colonization levels between bacterial isolates from plants compared to the bacterial isolate from seaweed and detected significant differences in colonization levels between phylogenetically similar bacteria. These findings demonstrate attachment qPCR can be used to quantify colonization levels of bacteria on plants with high resolution.

### 2.6. Bacterial Colonization of Lemna minor Visualized by Confocal Microscopy

**Figure 2:**
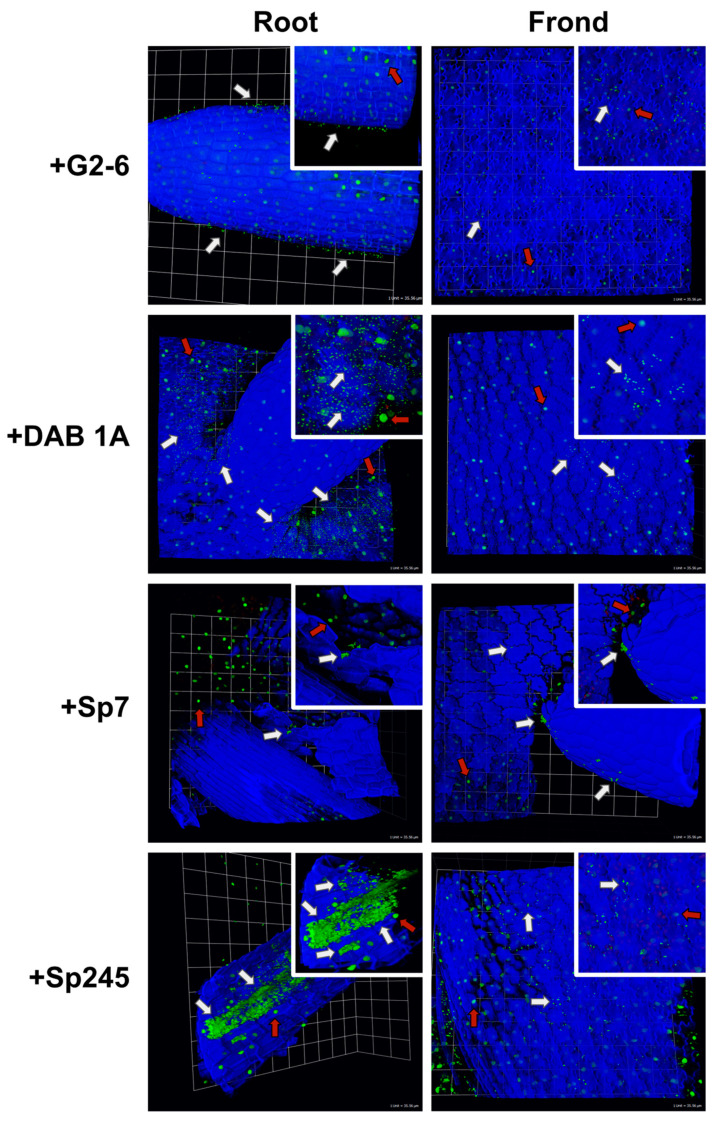
Two-dimensional confocal microscopy of inoculated duckweed samples. Confocal microscopy (40X/1.1 objective) was performed on inoculated Lm5576 in 0.5X SH media to characterize the spatial colonization dynamics of bacteria on duckweed. Calcofluor white was used to stain Lm5576 cellulose and visualized with the blue channel, whereas SYBR Gold was used to stain DNA and visualized with the green channel. Both bacterial and Lm5576 DNA were stained by SYBR Gold and pictured in green, but bacterial DNA (indicated by white arrows) is smaller in size than Lm5576 nuclei (indicated by red arrows), and these bacteria are often found in clustered colonies. For each main image, 1 unit of the grid scale is equal to 35.56 μm and zoomed-in images are pictured in the top-right corner. +G2-6 = Lm5576 inoculated with *Bacillus simplex* RUG2-6, +DAB 1A = Lm5576 inoculated with *Microbacterium* sp. RU370.1, +Sp7 = Lm5576 inoculated with *Azospirillum brasilense* Sp7, +Sp245 = Lm5576 inoculated with *Azospirillum baldaniorum* Sp245.

As a complementary approach to the PCR-based methods described above, confocal microscopy was performed on inoculated Lm5576 samples to qualitatively describe bacterial colonization patterns (Figure 2, File S5). Attachment PCR was performed on all microscopy samples and confirmed the colonization of Lm5576 by the respective bacteria and the absence of contaminating bacteria (File S6). All the bacteria tested were found to colonize the surfaces of Lm5576 fronds (Figure 2). G2-6, DAB 1A, and Sp245 were spread over the surfaces of Lm5576 fronds in smaller colonies, and Sp7 was mostly localized to the root–frond interface in aggregates. No bacteria were observed to colonize the inside of Lm5576 fronds in these experiments. Bacteria displayed different colonization patterns of Lm5576 roots (Figure 2, File S5). G2-6 was found on the surfaces of Lm5576 roots at a low density. As mentioned above, Sp7 aggregates were mostly located on the surfaces of Lm5576 roots near the root–frond interface. DAB 1A and Sp245 were also found mostly at the root–frond interface on the surfaces of Lm5576 roots. Microscopy showed DAB 1A was present in higher concentrations at the root–frond interfaces than the other bacteria tested. This correlates with the high colonization levels observed in attachment qPCR experiments for some samples (Figure 1B). Interestingly, Sp245 was found inside Lm5576 roots, within the endodermis, at high concentrations. This also agreed with the attachment qPCR results that revealed a significantly high colonization level for Sp245 and shows it is an endophyte for Lm5576. DAB 1A and Sp7 were also sporadically found inside Lm5576 roots but at much lower frequencies and concentrations. Together, confocal microscopy of inoculated Lm5576 samples revealed that the bacteria tested were able to colonize Lm5576 fronds and roots to various extents, and that the root–frond interface is a hotspot for bacterial colonization. In addition, these results support the main conclusions of the attachment qPCR experiments in this study (Figure 1B) while contributing to the understanding of spatial colonization dynamics of bacteria on duckweed tissues.

### 2.7. Strain-Specific Monitoring of Duckweed–Associated Bacteria in a Community Context

**Figure 3:**
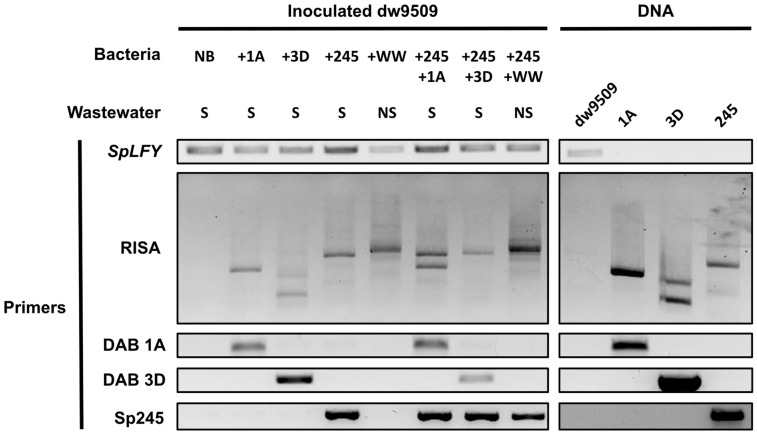
Molecular detection of duckweed-associated bacteria in a community context. Axenic dw9509 was inoculated with different bacteria in wastewater with (NS) or without (S) microbes. In addition, Sp245 was co-inoculated onto dw9509 with DAB isolates or non-sterile wastewater-containing microbes. For co-inoculated samples, bacteria were mixed at a 1:1 ratio based on OD_600_. After five days, dw9509 tissue was collected, washed with sterile water, and nucleic acids were isolated for end-point PCR using RISA, *SpLFY*, and strain-specific primers (File S2). RISA PCR fingerprints from inoculated dw9509 samples were compared to DNA controls from dw9509 and bacteria alone. Wastewater: S = filter-sterilized wastewater not containing microbes, NS = non-sterile wastewater containing microbes; Bacteria: NB = axenic dw9509, +1A = dw9509 inoculated with *Microbacterium* sp. RU370.1, +3D = dw9509 inoculated with *Bacillus* sp. RU9509.4, +Sp245 = dw9509 inoculated with *Azospirillum baldaniorum* Sp245, +WW = dw9509 inoculated with non-sterile wastewater containing microbes; Primers: *SpLFY* = PCR using SpLFY-F and SpLFY-R primers specific to dw9509, RISA = PCR using 16S-1390f and 23S-e130r primers, DAB 1A = PCR using AmRU370.1-F and AmRU370.1-R primers specific to *Microbacterium* sp. RU370.1, DAB 3D = PCR using BsRU9509.4-F and BsRU9509.4-R primers specific to *Bacillus* sp. RU9509.4, Sp245 = PCR using AbSp245-F and AbSp245-R primers specific to *Azospirillum baldaniorum* Sp245.

To further illustrate the efficacy of attachment PCR assay using bacterial strain-specific primers, this molecular approach was used to detect specific duckweed–bacterium associations in the presence of other bacterial isolates and in the presence of microbes in wastewater (Figure 3). Attachment PCR was also tested using another duckweed strain obtained from the RDSC, *Spirodela polyrhiza* strain 9509 (dw9509), whose genome was sequenced to reference quality [61,62]. For these experiments, axenic dw9509 was inoculated with DAB 1A, *Bacillus* sp. RU9509-4 (DAB 3D), Sp245, or with wastewater containing microbes (NS) for five days. In addition, Sp245 was co-inoculated onto dw9509 with DAB 1A, DAB 3D, or wastewater containing microbes (NS) to test bacterial colonization in the presence of other microbes. After five days, inoculated duckweed tissue was collected, rinsed with sterile water, and nucleic acids were isolated. *SpLFY* PCR generated a PCR product for all inoculated dw9509 samples, ensuring good sample quality. RISA PCR and strain-specific PCR did not generate any signals for axenic dw9509, confirming its sterility. RISA PCR showed DAB 1A, DAB 3D, Sp245, and wastewater microbes (NS) colonized dw9509. Additionally, strain-specific PCR confirmed DAB 1A, DAB 3D, and Sp245 colonized dw9509 while no amplification products were obtained with wastewater containing microbes sample (NS). RISA PCR and Sp245 strain-specific PCR demonstrated Sp245 was able to colonize dw9509 in the presence of DAB 1A, DAB 3D, and a wastewater microbial community, indicating robust colonization ability by Sp245 in diverse contexts. While both DAB 1A and DAB 3D were able to colonize dw9509 in the presence of Sp245, DAB 3D strain-specific PCR showed a lower amplification signal in the dw9509 sample co-inoculated with DAB 3D and Sp245 compared to the dw9509 sample inoculated only with DAB 3D, suggesting DAB 3D colonization, but not DAB 1A, of dw9509 was reduced in the presence of Sp245. These experiments illustrate the efficacy of attachment PCR and strain-specific PCR to detect specific duckweed–associated bacteria in a community context. In addition, quantitative effects on bacterial colonization of the plant host resulting from distinct microbe-microbe interactions can be revealed.

## 3. Discussion

### 3.1. Localization of Bacteria on Duckweed

In terrestrial plants, bacteria have been shown to consistently colonize certain areas of plants termed colonization hot spots [21,22], which include root cracks where lateral roots emerge from the main root [23]. One explanation for this bacterial colonization pattern is that root cracks may release cell lysates and exudates that could help attract bacteria and other microorganisms [63]. In duckweed, a few studies have described bacterial colonization patterns on these aquatic plants. In one study, duckweed collected from chalk streams was found to have higher densities of bacteria on the submerged abaxial surface of duckweed fronds compared to the aerial adaxial surface [64]. In another study on *L. minor*, the plant-growth-promoting bacterium, *Bacillus amyloliquefaciens* FZB42, was found to initially colonize *L. minor* at the root tip and root–frond interface, and later the grooves between root epidermal cells and concavities of the abaxial frond surface [29]. For a rootless duckweed, *Wolffia australiana*, bacteria present in the surrounding greenhouse environment were found to colonize near reproductive pockets, where mother and daughter fronds attach, and the stomata [4]. In the present study, we performed high-resolution confocal microscopy on inoculated *L. minor* samples to further study bacterial colonization patterns of duckweed (Figure 2). All bacteria in this study were able to associate with the abaxial surfaces of duckweed fronds and roots to varying extents. Like previous reports, bacterial strains used in this study also showed a preference for the root–frond interface. Together, these studies show that bacteria readily colonize the abaxial surface of duckweed fronds, at least for duckweeds with roots. One possible explanation for this observation is that the abaxial side of duckweed fronds is in direct contact with the microbial inoculum present in the surrounding water environment. Another explanation is that the surface composition, such as that of the cuticle, is distinct between the abaxial and adaxial surfaces of fronds [65] and may play a role in the differential attachment of microbes. Although duckweeds do not make lateral roots [66], the root–frond interface in duckweed may serve as a hotspot akin to the root cracks in terrestrial plants, where cell contents are released or secreted to attract microbes.

While the surface colonization patterns of bacteria on duckweed have been described, there is no description of whether bacteria can colonize the inside of duckweed tissues. In this study, Sp245 colonized Lm5576 at the root–frond interface and the inside of duckweed roots, within the endodermis [66], at high densities (Figure 2). In terrestrial plants, colonization hot spots, such as root cracks, can be used by bacteria to enter the roots of terrestrial plants [23,67,68]. Likewise in duckweed, one possibility could be that Sp245 entered through cracks at the root–frond interface of Lm5576 and proceeded to colonize the interior of Lm5576 roots. Recently, we studied the Sp245 interaction with the model plant *Arabidopsis thaliana* and revealed its potential interaction with guard cells in leaf tissues as a means for entering the endosphere [55]. Strikingly, this interaction and targeting to the guard cells by Sp245 are abolished in the pleiotropic *axr1* mutant of *A. thaliana*, suggesting specific involvement of this gene in the signaling between plants and certain microbes. However, our microscopy studies with Lm5576 failed to observe this guard-cell colonization of Sp245 on duckweed fronds, indicating this mode of interaction could be lost or modified in duckweed. Sp245 was originally isolated from wheat experiments in Brazil [69] and colonizes the inside of wheat roots at high densities [24,26]. In contrast to the endophytic colonization pattern of Sp245, Sp7, originally isolated from *Digitaria* in Brazil [70], is an epiphyte shown to colonize only the surfaces of plant roots such as wheat and corn [24,26,71]. In particular, Sp7 has been found to form aggregates on the surface of these plant roots. Likewise, we found Sp7 mostly colonized the surface of Lm5576 roots near the root–frond interface, also in aggregates. These Sp7 and Sp245 colonization experiments on Lm5576 demonstrated that plant-associated bacteria can have similar colonization patterns with both terrestrial plants, such as wheat, and aquatic monocots, such as duckweed. This suggests a likely conservation of bacterial mechanisms for associating with duckweed and other higher plants.

### 3.2. Molecular Methods for Detection of Duckweed-Associated Bacteria

Classical microbiology techniques, such as the CFU assay, have been used to monitor bacterial colonization of duckweed [72]. As mentioned above, this CFU assay lacks the specificity to differentiate bacteria within a community context. More recent studies have demonstrated the ability of a molecular approach using bacterial primers and real-time PCR to quantify bacterial colonization levels on duckweed in the presence of other microbes [73,74]. In this study, we built on this molecular approach and developed different PCR-based strategies to detect duckweed-associated bacteria. A prerequisite for using such approaches is a protocol capable of efficiently isolating nucleic acids. Thus far, there have been no published attempts to systematically develop a protocol capable of efficiently isolating nucleic acids from both duckweed and bacteria. A working protocol for isolating nucleic acids is critical for studying duckweed–bacteria associations, since different DNA extraction protocols can introduce significant biases toward what bacteria are detected and in what quantities [75,76]. These differences can be partly explained by the inability of certain protocols to efficiently lyse monoderm bacteria, Gram-positive bacteria consisting of a thick peptidoglycan layer. However, nucleic-acid-isolation protocols implementing physical lysis methods such as bead-beating can efficiently lyse monoderm bacteria, especially when longer bead-beating times are used [77,78,79]. Bead-beating protocols are also reproducible [76,80], yield high concentrations of nucleic acid [76,81], and produce relatively accurate community profiles [82]. In addition, combining bead-beating and chemical lysis, such as phenol or chloroform, can dramatically increase DNA extraction efficiency and quality [76,81]. For these reasons, bead-beating is recommended for nucleic acid extraction protocols [83]. Here, we implemented and optimized a bead-beating protocol to simultaneously co-isolate duckweed and bacterial nucleic acids. By combining different bead sizes and a CTAB/chloroform lysis buffer, this bead-beating protocol produced high yields of intact nucleic acids from duckweed and different bacteria, including monoderm and diderm bacteria (Figures S2–S5). Furthermore, through various testing of the bead-beating protocol, we observed increases in nucleic acid yields with longer incubation time periods in the lysis buffer (Figure S5) and with longer alcohol precipitation time periods. However, the ability of this bead-beating protocol to generate representative profiles of duckweed colonized by complex bacterial communities remains to be validated. This could be tested by isolating nucleic acid from mixtures of bacteria in known concentrations, known as mock communities [84,85]. This will be an important validation step for applying this bead-beating protocol to study the interactions between duckweed and complex microbial communities in the future. Lastly, this bead-beating protocol can be modified to isolate only DNA or RNA from duckweed or bacteria by adding an RNase or DNase treatment step, respectively.

rRNA intergenic spacer analysis (RISA) has been commonly used for community fingerprinting [59] and bacterial typing [60]. However, RISA has also been used to study plant–bacteria associations. For example, an automated version of RISA (ARISA) that applies fluorescently tagged primers and detects fluorescent PCR fragments [86] was used to monitor changes in the composition of synthetic bacterial communities [87]. Here, we used RISA to detect bacterial colonization of duckweed by comparing fingerprints of inoculated duckweed samples to DNA controls of duckweed and the respective bacteria (Figure 1A and Figure 3). This molecular approach serves many purposes. First, RISA can be used to determine the axenic status of *L. minor* and *S. polyrhiza* plants used in experiments, since RISA PCR does not produce any amplicons from sterile Lm5576 and dw9509. This is worth highlighting since difficulties can be encountered in obtaining sterile duckweed [88]. As we optimized RISA PCR for use with *L. minor* and *S. polyrhiza* in this study, RISA PCR may need to be optimized for use with other duckweed species by using different RISA primer sets and/or PCR conditions. Second, RISA can be used to determine the colonization of duckweed by different bacterial genera, since RISA PCR can produce distinct fingerprints between bacterial genera. This is particularly useful when no sequence information is available to design primers for the bacteria being studied. Third, because fingerprints generated from inoculated duckweed samples are compared to DNA controls of the organisms being studied, RISA can also reveal non-matching fingerprints that are due to contaminating or exogenous bacteria during the experiment. In addition to RISA, we included a duckweed-specific marker to control for sample quality and to provide a reference for the relative quantity of duckweed DNA between samples. We termed this approach of combining RISA PCR and PCR of a plant-specific marker as attachment PCR. Attachment PCR was recently used in our laboratory to detect interactions between bacteria isolated from rice and duckweed [89]. Under laboratory conditions, this method showed that *Pantoea* isolates from rice were able to colonize duckweed such as Lm5576, despite the low representation of *Pantoea* bacteria in community surveys of duckweed-associated bacterial (DAB) communities from the same rice paddies. This suggests that microbe-microbe interactions or environmental factors could be responsible for the low representation of *Pantoea* in DAB communities, but not in rice tissues, in this context. These case studies demonstrate the utility of RISA in general and its application for attachment PCR specifically.

Despite its advantages, we found RISA was unable to distinguish between strains from the same genus (Figure 1A). As mentioned previously, plant-associated bacteria from the same genus can be functionally diverse. Thus, it is important to identify methods that can differentiate closely related bacteria isolated from plants. Previous studies have used strain-specific primers to monitor bacteria associated with duckweed [73,74]. However, these studies did not explicitly define the primer design strategy and did not verify whether the primers used could differentiate phylogenetically similar bacteria. Here, we used available bioinformatic tools and developed a computational pipeline (UniAmp) to generate strain-specific primers, leveraging the large and growing databases for bacteria (Figure S8, Files S2 and S4). Strain-specific primers for end-point PCR were able to clearly differentiate strains from the same genus (Figure 1A) and detected specific bacteria even in the presence of other microbes (Figure 3). Strain-specific primers were also designed for real-time PCR to determine colonization levels of specific bacteria on duckweed by normalization to a duckweed-specific reference marker (Figure 1B). This “attachment qPCR” approach, of normalizing bacterial abundance to an internal plant marker gene, has been applied in previous studies to quantify maize root colonization by plant-growth-promoting bacteria (PGPB) [48], *Petunia* root colonization by arbuscular mycorrhizal fungi [90], and *Arabidopsis* root colonization by bacteria belonging to the Rhizobiales family [91]. For all these uses of strain-specific primers to detect bacterial colonization of different plants, many studies do not provide an accessible primer design strategy. In a few of the remaining studies, a primer design strategy is used that splits bacterial genome sequences into smaller sequence fragments that are then used for a BLAST similarity search against public databases to find unique sequences for primer design [48,49]. Yet, this primer design strategy can be inefficient if many strain-specific primers are desired or if large genomes are being used. Here, we developed a straightforward computational pipeline (UniAmp) to design strain-specific primers for any bacterial strain with a sequenced genome. In this pipeline, pairwise genome alignments are performed between a reference genome for the bacterial strain of interest and a selected set of query genomes. By retrieving a set of query genomes from public databases that are highly similar to the reference genome, unique sequences to the reference genome can be determined and used for primer design. Due to the efficiency of pairwise genome alignments [92], this approach can be readily applied to design strain-specific primers for many genomes or for large genomes. While the UniAmp pipeline was used in this study to design primers to detect bacteria associated with duckweed, the pipeline could be readily applied to other host systems and many other scenarios.

### 3.3. Bacterial Adaptation to Plant Habitats and Colonization Dynamics

Selection is a major driver in structuring plant bacterial communities [2]. As a result of this selection, certain bacteria have adapted to occupy different plant habitats [93,94]. For example, genomic analyses have shown that plant-associated bacterial genomes are enriched in certain functions, such as chemotaxis, motility, and carbohydrate metabolism [95,96]. In support of these analyses, genome-wide functional screens using transposon mutagenesis sequencing in both terrestrial plants and duckweed confirm the involvement of chemotaxis, motility, and carbon metabolism in bacterial colonization of plants [97,98]. In addition to these functions, many plant-associated bacteria can produce phytohormones, such as auxins, which can have either beneficial or detrimental effects on plant hosts [99]. Most studies on bacterial auxin production have focused on the effects on plant growth, but one recent study investigated the role of bacterial auxin production in plant colonization [100]. This study showed that auxin production is necessary for some bacteria to efficiently colonize plant roots and revealed a feedback loop between auxin produced by bacteria and reactive oxygen species (ROS) produced by the plant host. Together, these studies describe some of the functions that may have evolved in bacteria to help colonize plants.

In this study, we explored the colonization levels among a non-plant-associated bacterial isolate and several plant-associated bacterial isolates using attachment qPCR. G2-6 was isolated from seaweed, a macroalga from salt water, and likely has not adapted or evolved to colonize freshwater macrophytes such as duckweed. We thus expected G2-6 to colonize duckweed at very low levels, if at all. Indeed, attachment qPCR showed G2-6 associated with Lm5576 at significantly lower concentrations compared to all the plant-associated bacterial isolates tested (Figure 1B). DAB 1A was originally isolated from Lm5576 and produces high levels of the auxin indole-3-acetic acid (IAA) that can affect the root development of *Arabidopsis thaliana* [54,55]. Therefore, we expected DAB 1A to re-colonize Lm5576 in this study. Confocal microscopy confirmed these expectations and showed high levels of DAB 1A near the root–frond interface of Lm5576 (Figure 2). Attachment qPCR showed variable colonization levels, where DAB 1A colonized Lm5576 at high levels in some samples (Figure 1B). Extending these results, confocal microscopy of *A. thaliana* inoculated with DAB 1A showed high concentrations of DAB 1A present on the root surface [55]. Interestingly, this same study showed another DAB isolate, DAB 33B, was not able to colonize *A. thaliana*, even though it belonged to the same genus, *Microbacterium*, as DAB 1A. In addition to this inability to colonize *A. thaliana*, DAB 33B was shown to produce significantly lower levels of IAA compared to DAB 1A. Together, one explanation for the different colonization dynamics between these phylogenetically similar strains (DAB 1A and DAB 33B) could be that high levels of auxin production facilitate DAB 1A colonization of plants. Members of the *Azospirillum* genus are well-known plant colonizers and have been shown to fix nitrogen and produce phytohormones, such as IAA, which may promote plant growth [56]. Interestingly, *Azospirillum* taxa have also been detected in surveys of DAB communities [3] and isolated from duckweed tissues [101]. Therefore, we hypothesized that the *Azospirillum* strains Sp7 and Sp245 would be able to colonize Lm5576 to some extent. Attachment qPCR showed Sp245 colonized Lm5576 at significantly higher levels than Sp7 (Figure 1B). This was further supported by confocal microscopy, which showed high levels of Sp245 within duckweed roots (Figure 2). These results are similar to a previous study that found Sp245 colonized the root endosphere of wheat and contained higher overall colonization levels compared to Sp7 [24].

Together, these attachment qPCR results raise several implications about the bacterial colonization of plants. For one, these data suggest that bacteria adapted to plants may display significantly higher colonization levels compared to non-adapted bacteria (Figure 1B). If so, then attachment qPCR can be used to screen for bacteria adapted to colonizing plant habitats. This kind of experiment may help to discover novel traits necessary for the successful bacterial colonization of plants. Secondly, the plant-associated bacterial isolates examined in the present work showed different colonization levels. This raises the question of what traits determine the colonization levels of bacteria on plants? As mentioned above, auxin production is necessary for some bacteria to colonize plants [100]. Interestingly, the plant-associated bacterial isolates DAB 1A, Sp7, and Sp245 all produce significant levels of auxin [54,55]. Future work could use attachment qPCR to examine the relationship between the level of bacterial auxin produced and the effect on bacterial colonization level of the plant host. Results from this study also showed significantly higher colonization levels for the endophyte Sp245 compared to the epiphyte Sp7 (Figure 1B). This also raises the question of what is the relationship between bacterial colonization levels and bacterial colonization patterns? Do all endophytes display high colonization levels? If not, what controls the colonization levels of different endophytes? To answer this, attachment qPCR experiments could be performed to quantify the colonization levels of different bacterial endophytes. In summary, quantitative studies using attachment qPCR could lead to an improved understanding of traits underlying bacterial colonization of plants.

### 3.4. Molecular Detection of Specific Duckweed–Associated Bacteria within a Community Context

Similar to findings with terrestrial plant bacterial communities, microbe–microbe interactions likely play an important role in the bacterial colonization of duckweed. One study reported that a PGPB and two different plant-growth-inhibiting bacteria (PGIB) showed stable colonization levels of duckweed when inoculated separately [73]. However, when the PGPB and PGIB were co-inoculated together, the PGPB strain completely excluded one of the PGIB from colonizing duckweed. In another study, the same PGPB strain slowly decreased in abundance over time on duckweed in the presence of different bacterial communities [74]. Thus, the ability to distinguish between phylogenetically similar microbes in both mono-associations and within a community context will be important for studying the interactions between plants and complex microbial communities. In our work, strain-specific primers were shown to detect specific duckweed–associated bacteria within a community context (Figure 3). The specificity demonstrated by strain-specific PCR has a pertinent application in the synthetic ecology approach used to study plant–microbe interactions [102]. In this approach, synthetic bacterial communities (SynComs) are constructed from bacterial isolates that are representative of members found in wild plant bacterial communities. In contrast to wild bacterial communities, SynComs are experimentally amenable and tractable, allowing causal relationships to be determined. As a constructed community, SynComs can capture the complexity of plant bacterial communities found in nature while providing a means to decipher mechanisms underlying community dynamics and functions [20]. However, SynComs are limited by methods commonly used to track member presence and abundance, such as 16S rDNA amplicon sequencing. Since 16S rDNA amplicon sequencing cannot distinguish between many phylogenetically similar bacteria, SynComs have to be carefully designed in a way to select distinguishable members [103]. As a result, this can severely limit the diversity and representativeness of SynComs that can be used to effectively study the colonization dynamics of plant microbial communities. However, using strain-specific primers will allow closely related bacteria to be included and monitored in SynCom experiments. Moreover, attachment qPCR can be used to quantify member abundance in SynCom experiments. The strategy used in attachment qPCR where bacteria load is normalized to the quantity of host DNA is similar to traditional qPCR used in RNA-sequencing experiments to validate gene expression, where a target gene is normalized to a housekeeping gene. In an analogous fashion, attachment qPCR could be used to compare member abundance generated from 16S rDNA amplicon sequencing in SynCom experiments, since both approaches are DNA-based. Moreover, attachment qPCR could allow phylogenetically similar bacteria with different colonization dynamics and functional traits to be used in SynComs. Such comparisons could facilitate the assignment of the different phenotypes observed in SynCom experiments to specific molecular features. Together, these kinds of experiments should facilitate a mechanistic understanding of the interactions between plant hosts and their associated microbes.

## 4. Conclusions

In conclusion, the PCR-based approaches introduced in this study have been shown to be effective for studying duckweed–associated bacteria. Attachment PCR with generic RISA primers can be used to reveal the bacteria associated with duckweed, and PCR using strain-specific primers can be used to differentiate specific duckweed–bacterium associations. Additionally, the attachment qPCR approach can be used to determine colonization levels of bacteria under binary or community contexts. While these molecular approaches were used to study duckweed–associated bacteria in this study, they should be easily adopted for use with other host-microbe systems. Together, these strain-specific approaches overcome the limitations of current methods used to detect plant–bacteria associations and enable the detection and quantification of specific plant–microbe associations under diverse scenarios.

## 5. Materials and Methods

### 5.1. Duckweed Sterilization and Propagation

Cultures of *Lemna minor* 5576 (Lm5576) and *Spirodela polyrhiza* (dw9509) were obtained from the Rutgers Duckweed Stock Cooperative (RDSC; Rutgers University, New Brunswick, NJ, USA). Duckweed cultures were sterilized using a modified protocol from a previously described procedure [88]. For this procedure, duckweed plants were transferred to 1.7 mL microcentrifuge tubes and washed with 500 µL of salt and detergent solution (1% Triton-X 100, 137 mM NaCl, 2.7 mM KCl, 10 mM Na_2_HPO, 1.8 mM KH_2_PO_4_, 0.5 mM MgSO_4_, 1 mM CaCl_2_, pH 7.4) to facilitate surface sterilization. Duckweed plants were then surface sterilized using 5–10% (*v/v*) household bleach (0.5–1% sodium hypochlorite). Duckweed plants were surface sterilized until most frond tissues turned white, and only the meristematic regions retained chlorophyll and remained green. Following surface sterilization, 2% (*w/v*) of sodium thiosulfate was added to help neutralize residual sodium hypochlorite [104]. Surface-sterilized duckweed plants were then rinsed with sterile water and aseptically transferred to 0.8% (*w/v*) agar (BD, Franklin Lakes, NJ, USA; catalog #214530) plates with 1.6 g/L of Schenk and Hildebrandt basal salt mixture (0.5X SH) media (Phytotechnology Laboratories, Lenexa, KS, USA; catalog #S816) containing 0.5% sucrose and 100 μg/mL cefotaxime (GoldBio, St. Louis, MO, USA; catalog #C-104-25) at pH 6.5–7.0. In addition, surface-sterilized duckweed plants were transferred to 1.5% (*w/v*) agar plates with Miller’s lysogeny (or Luria) broth (LB) (10 g/L tryptone, 5 g/L yeast extract, 10 g/L NaCl). Surface-sterilized duckweed plants were propagated on 0.5X SH agar plates and a LB agar plate. The LB agar plate was used to detect any signs of microbial growth. If microbial growth was observed on duckweed plants growing on the LB agar plate, then the surface-sterilization procedure was repeated on the surface-sterilized duckweed plants growing on the 0.5X SH agar plates.

Once axenic duckweed plants were obtained, stock cultures and working cultures of axenic duckweed were generated. Stock cultures of axenic duckweed were stored at 15 °C and only used when required. Axenic working cultures of duckweed were generated by transferring a few duckweed plants to a 0.5X SH agar plate with 0.5% (*w/v*) sucrose and an LB agar plate after each experiment. If no microbial growth was observed on the LB agar plate, then duckweed plants on the 0.5X SH agar media were propagated for experiments. If microbial growth was observed, then a stock culture of axenic duckweed was retrieved from storage and propagated for experiments.

Axenic duckweed plants were propagated in a growth chamber on 0.5X SH agar media with 0.5% (*w/v*) sucrose (pH 6.5–7.0) at 25 °C under a photoperiod of 16 h light and 8 h dark for 2–4 weeks. Duckweed plants from the agar plate were then transferred to 100 mL of liquid culture of 0.5X SH with 0.1% (*w/v*) sucrose and propagated for 1–2 weeks under the same growth chamber conditions. Axenic duckweed plants from these liquid cultures were then transferred for experiments. Duckweed sterility was confirmed between transfers by plating duckweed plants on LB agar plates and checking for microbial growth.

### 5.2. Isolation and Identification of Bacteria

To inoculate duckweed with bacteria for experiments, bacteria were isolated from different duckweed samples and the seaweed *Ulva fasciata* by washing tissues before homogenization and plating on LB agar or tryptic soy agar (TSA; BD, Franklin Lakes, NJ, USA; catalog #236950) plates at 28 °C for up to three days (File S1). For some bacterial isolates, plant host tissues were surface sterilized, using the procedure described above, before isolation. Pure cultures for these bacterial isolates were generated by picking single colonies from LB agar or TSA plates and inoculating liquid LB or tryptic soy broth (TSB; Hardy Diagnostics, Santa Maria, CA, USA; catalog #C7141) for up to two days at 28 °C. Glycerol stocks were then generated for each isolate and stored at –80 °C as stock cultures until further use. Cultures of *Azospirillum* strains, *A. brasilense* Sp7, and *A. baldaniorum* Sp245 (formerly *A. brasilense*) [57] were obtained from S. Lebeis (MSU) and stored as glycerol stocks.

Bacterial isolates from duckweed and seaweed were previously identified using the following procedure [54]. The 16S rRNA gene fragment was amplified with polymerase chain reaction (PCR) using the primers 16S-e9f and 16S-e926r (File S2) [32]. PCR reactions were prepared in a total volume of 25 uL consisting of: 1X PCR buffer with Mg^2+^ (1.5 mM MgCl_2_, 10 mM KCl, 8 mM (NH_4_)_2_SO_4_, 10 mM Tris-HCl, pH 9.0, 0.05 % NP-40; Thomas Scientific, Swedesboro, NJ, USA), 0.4 μM forward primer, 0.4 μM reverse primer, 0.2 mM dNTPs, 2 units of Choice-Taq DNA polymerase (Thomas Scientific, Swedesboro, NJ, USA; catalog #CB4050-2), and 1 μL of either bacterial nucleic acid (100 ng/μL), bacterial DNA (5 ng/μL), or bacterial liquid culture. PCR reactions were performed using the following 3-stage thermocycler program: (1) denaturation stage of 95 °C for 5 min; (2) 25 cycles consisting of 95 °C for 30 s, 50 °C for 30 s, and 72 °C for 1 min; and (3) a final extension stage of 72 °C for 5 min. PCR products were cleaned using ExoSAP-It PCR Product Cleanup Reagent (ThermoFisher Scientific, Waltham, MA, USA; catalog #78200.200.UL) or DNA Clean and Concentrator-5 kit (Zymo Research, Irvine, CA, USA; catalog #D4003). PCR products were sent to Genewiz (South Plainfield, NJ, USA) for sequencing using both 16S-e9f and 16S-e926r primers.

For each isolate, the resulting chromatograms for both forward and reverse sequences were analyzed, and poor-quality sequences at both 5′ and 3′ ends were cropped using FinchTV v1.3.0 (Geospiza, Inc.) (www.digitalworldbiology.com). Forward and reverse sequences were aligned using SerialCloner v2.6.1 (http://serialbasics.free.fr/Serial_Cloner.html) to generate a consensus sequence. Gaps and mismatches were corrected in the consensus sequence using the chromatograms of the raw sequences. The consensus sequence was cropped 216 bp downstream and 385 bp upstream of the conserved U515 (5′-GTGCCAGCAGCCGCGGTAA-3′) sequence [32] to generate a 620 bp processed sequence. Processed sequences were annotated using the RDP classifier v2.13 with the 16S rRNA training set 18 [105].

### 5.3. Genome Sequencing of Bacterial Isolates

Draft genomes were generated at the DOE Joint Genome Institute (JGI) for duckweed-associated bacterial (DAB) isolates DAB 1A and DAB 3D and the seaweed bacterial isolate G2-6 (File S1). Standard 300 bp Illumina shotgun libraries were constructed for all isolates.

DAB 1A (*Microbacterium* sp. RU370.1) and DAB 3D (*Bacillus* sp. RU9509.4) libraries were sequenced with the Illumina HiSeq 2000 platform. Raw reads were filtered for artifacts using BBDUK (Bushnell B., sourceforge.net/projects/bbmap/). Filtered reads were assembled using Velvet v1.2.07 [106] with the following parameters for velveth: 63, -shortPaired and the following parameters for velvetg: -very_clean yes, -exportFiltered yes, -min_contig_lgth 500, -scaffolding no, -cov_cutoff 10. Velvet contigs were then used to create 1–3 kb simulated paired end reads using wgsim v0.3.0 (https://github.com/lh3/wgsim) with the following parameters: -e 0, -1 100, -2 100, -r 0, -R 0, -X 0. Simulated read pairs were then used to assemble Illumina reads using Allpaths-LG version r46642 [107] with the following parameters: PrepareAllpathsINputs, RunAllpathsLG. Assembly of 16S rRNA genes (percent 16S rRNA sequence covered in assembly is ≥80% or length ≥ 1000 bp) was performed using filtered Illumina reads and non-duplicated sequences were merged into Allpaths assembly.

G2-6 (*Bacillus simplex* RUG2-6) libraries were sequenced with the Illumina HiSeq-2500 1TB platform. Read were processed using the BBTools suite at JGI (BBMap—Bushnell B.—sourceforge.net/projects/bbmap/). Raw reads were filtered for artifacts using BBDUK based on the following criteria: more than one N, quality scores on average less than 8 (before trimming), or reads shorter than 51 bp (after trimming). Reads were then mapped to masked versions of human, cat, and dog references and discarded if identity was greater than 95% using BBMap. Reads were then masked using BBMask. Processed reads were assembled using SPAdes v3.6.2 [108] with the following parameters: –cov-cutoff auto, –phred-offset 33, -t 8, -m 40, –careful, -k 25,55,95, –12. Assembly contigs less than 1 kbp were discarded.

### 5.4. Inoculation of Duckweed with Bacteria

To study the bacterial colonization of duckweed, axenic duckweed was inoculated with the bacterial isolates described above. To inoculate duckweed with bacteria, a glycerol stock for the respective bacterium was used to inoculate a 5 mL liquid culture of LB or TSB and grown for up to two days at 28 °C by shaking on a rotating platform at 220 rpm. A volume of 500 μL from the 5 mL liquid culture was then used to inoculate a 50 mL liquid culture of LB or TSB and grown for up to two days at 28 °C at 220 rpm. The 50 mL bacterial culture was spun at 8000 rpm for 5 min at 4 °C. The supernatant was then decanted, and the bacterial pellet was resuspended and washed with 0.5X SH. The sample was centrifuged as mentioned above. The resulting bacterial pellet was resuspended in 0.5X SH media and diluted to an optical density (600 nm) of 0.2 in a final volume of 50 mL in a glass plant tissue culture vessel (Phytotechnology Laboratories, Lenexa, KS, USA; catalog #C1770). Duckweed was then transferred to this 50 mL bacterial culture to cover the entire surface of the 50 mL bacterial culture. Inoculated duckweed was then incubated in a growth chamber under the same conditions used for duckweed propagation described above.

Wastewater samples were used to examine the colonization of duckweed by bacterial isolates in the presence of a microbial community. Wastewater samples were collected from the United Water Princeton Meadows wastewater treatment facility (Plainsboro, NJ, USA) after secondary clarification. For wastewater experiments, duckweed was inoculated as described above in 50 mL of non-sterile or filter-sterilized wastewater using 0.2 μm polyethersulfone filters.

### 5.5. Nucleic Acid Isolation from Duckweed and Bacteria

A bead-beating protocol was used to isolate nucleic acid from duckweed and bacteria. A combination of one 4 mm glass bead (OPS Diagnostics, Lebanon, NJ, USA; catalog #BAWG 4000-200-18), 0.5 g of 1.7 mm zirconium beads (OPS Diagnostics, Lebanon, NJ, USA; catalog #BAWG 1700-300-22), and 0.5 g of 100 μm silica beads (OPS Diagnostics, Lebanon, NJ, USA; catalog #BAWG 100-200-10) was used for bead-beating to lyse samples. The lysis buffer consisted of 300 μL of high salt CTAB buffer (100 mM Tris-HCl pH 8, 2.0 M NaCl, 20 mM EDTA, 2% CTAB) and 300 μL chloroform. Duckweed, bacteria, or inoculated duckweed samples were transferred to bead-beating tubes with beads and lysis buffer and homogenized using an HT6 benchtop homogenizer from OPS Diagnostics (Lebanon, NJ, USA) for 5 min (10 cycles of 30-s homogenization and 10-s pause) at 4000 rpm at room temperature or 4°C. The remaining steps were carried out at room temperature. After homogenization, samples were then centrifuged at 16,000× *g* for 5–10 min. Supernatants were transferred to new tubes and washed with 1× volume of 24:1 chloroform:isoamyl alcohol to remove protein precipitate and centrifuged at 16,000× *g* for 5–10 min. This wash step was repeated. Supernatants were then transferred to new tubes and 0.5× volume of 7.5 M ammonium acetate and 2.5× volume of 95% chilled ethanol were added [109]. Samples were centrifuged at 16,000× *g* for 30 min to pellet the precipitated nucleic acid. The resulting sample pellets were washed with 70% chilled ethanol and centrifuged at 16,000× *g* for 5–10 min. This step was repeated. Sample pellets were then air-dried and resuspended in 20 μL of sterile water or TE buffer. Nucleic acid concentration of samples were measured with a NanoDrop microvolume spectrophotometer (ThermoFisher Scientific, Waltham, MA, USA).

### 5.6. Molecular Detection of Duckweed-Associated Bacteria by rDNA Intergenic Spacer Analysis (RISA)

Primers for rRNA intergenic spacer analysis (RISA) were designed using previous reports (File S2) [32,59,60,86]. The primers 16S-e1390f and 23S-e130r were selected to detect bacterial colonization of Lm5576 (File S2). RISA PCR reactions were prepared in a total volume of 25 μL consisting of: 0.5 mM MgCl_2_, 1X PCR buffer with Mg^2+^ (1.5 mM MgCl_2_, 10 mM KCl, 8 mM (NH_4_)_2_SO_4_, 10 mM Tris-HCl, pH 9.0, 0.05% NP-40; Thomas Scientific, Swedesboro, NJ, USA), 0.2 mM dNTPs, 0.8 μM forward primer, 0.8 μM reverse primer, 2.5 units of ChoiceTaq DNA polymerase (Thomas Scientific, Swedesboro, NJ, USA; catalog # CB4050–2), and 2 μL of either nucleic acids from inoculated duckweed (~100 ng/µL) or bacterial DNA (~5 ng/μL). RISA PCR reactions were executed using the following 3-stage thermocycler program: (1) denaturation stage of 95 °C for 5 min; (2) 30 cycles consisting of 95 °C for 15 s, 60 °C for 30 s, and 72 °C for 1 min 30 s; and (3) a final extension stage of 72 °C for 5 min. RISA PCR products were visualized on a 1.0% (*w/v*) agarose gel stained with ethidium bromide.

To verify sample quality and the relative amount of duckweed DNA in samples, primers were designed for the single-copy, plant-specific *LEAFY* gene (File S2). *LEAFY* gene (*LFY*) primers were designed for dw9509 [62] and *L. minor* 5500 (Lm5500) [110]. Assembly and annotation files were retrieved from CoGe (https://genomevolution.org/coge/) for dw9509 (id 51364) and Lm5500 (id 27408). The LEAFY protein from *Arabidopsis thaliana* (NP_200993.1) was searched against the proteomes of dw9509 and Lm5500 using BLASTP v2.10.0+ [111]. Gene sequences were retrieved for top hits and a pairwise global alignment was generated using MUSCLE v3.8 [112]. The primers LmLFY-F and LmLFY-R were used to amplify the *LEAFY* gene from Lm5576 for endpoint PCR (*LmLFY* PCR) (File S2). The primers SpLFY-F and SpLFY-R were used to amplify the *LEAFY* gene from dw9509 for endpoint PCR (*LmLFY* PCR) (File S2). The primers qLFY-F and qLFY-R were used to amplify the *LEAFY* gene from Lm5576 for real-time PCR (File S2). *LmLFY* and *SpLFY* PCR reactions were prepared in a total volume of 25 μL consisting of: 1× PCR buffer with Mg^2+^ (1.5 mM MgCl_2_, 10 mM KCl, 8 mM (NH_4_)_2_SO_4_, 10 mM Tris-HCl, pH 9.0, 0.05% NP-40; Thomas Scientific, Swedesboro, NJ, USA), 0.2 mM dNTPs, 0.4 μM forward primer, 0.4 μM reverse primer, 2 units of ChoiceTaq DNA polymerase (Thomas Scientific, Swedesboro, NJ; catalog # CB4050-2), and 2 μL of either nucleic acids from inoculated duckweed (~100 ng/μL) or duckweed DNA (~5 ng/μL). *LmLFY* PCR reactions were executed using the following 3-stage thermocycler program: (1) denaturation stage of 95 °C for 5 min; (2) 28 cycles consisting of 95 °C for 15 s, 60 °C for 15 s, and 72 °C for 45 s; and (3) a final extension stage of 72 °C for 5 min. *SpLFY* PCR reactions were executed using the following 3-stage thermocycler program: (1) denaturation stage of 95 °C for 5 min; (2) 26 cycles consisting of 95 °C for 15 s, 60 °C for 15 s, and 72 °C for 30 s; and (3) a final extension stage of 72 °C for 5 min. *LmLFY* and *SpLFY* PCR products were visualized on a 1.0% (*w/v*) agarose gel stained with ethidium bromide.

### 5.7. Confocal Microscopy of Lm5576 Colonized by Bacteria

Lm5576 was inoculated with bacteria, as described above. After seven days, inoculated Lm5576 tissue was harvested, washed with sterile H_2_O, and fixed in 1 mL of 4% paraformaldehyde at room temperature in the dark overnight. The following day, the fixative solution was decanted, and the fixed tissue was washed with 1 mL of 1× phosphate-buffered saline (PBS) twice. Fixed tissue was then stored at 4 °C in 1 mL 1× PBS until further processing.

For confocal microscopy, paraformaldehyde fixed Lm5576 plants were gently washed in 1× PBS and stained for DNA 16 h at 4 °C with SYBR Gold nucleic acid stain (ThermoFisher Scientific, Waltham, MA, USA) diluted 1000 times in 1× PBS. Samples were then washed with 1× PBS and stained with 0.5 mg/mL calcofluor white stain (Sigma-Aldrich, St. Louis, MO, USA) for cellulose for 10 min at 22 °C. Confocal images were acquired at 1 µm z-steps on a Zeiss LSM 710 (Carl Zeiss MicroImaging GmbH, Jena, Germany) scanning head confocal microscope with a Zeiss plan apo 40X/1.1 objective. Excitation lasers were 405, 488, and 633 nm for the blue, green, and red emission channels, respectively. The calcofluor white fluorescence was detected at 410–551 nm, SYBR Gold fluorescence was detected at 533–572 nm, and chlorophyll autofluorescence was detected at 680–721 nm. Laser dwell times were 2.55 µs for all channels. Image analysis (2D and 3D) was conducted using Zen (Zeiss) or Volocity (PerkinElmer, Waltham, MA, USA). Both duckweed DNA and bacteria DNA were stained by SYBR Gold. Two-dimensional confocal Z-plane image stacks were visually analyzed to distinguish Lm5576 nuclei from stained bacteria cells based on their sizes and patterns of localization.

### 5.8. Strain-Specific Primer Design and End-Point PCR

Strain-specific primers were designed to detect the colonization of duckweed by specific bacterial strains (File S2). Two approaches were used to design primers; both approaches required sequenced genomes of the respective bacterial strains. The first approach used Panseq v3.2.1 [113] to find unique sequences for primer design for endpoint PCR. The following configuration settings were used: minimumNovelRegionSize 500, novelRegionFinderMode unique, fragmentationSize 1000, percentIdentityCutoff 85, and coreGenomeThreshold 2, runMode novel. The resulting unique sequences were then used for primer design. Primers were designed using the Primer3Plus web interface [114] with the following general settings: Primer Size Min 18, Primer Size Opt 20, Primer Size Max 25, Primer Tm Min 57, Primer Tm Opt 60, Primer Tm Max 63, Primer GC% Min 40, Primer GC% Opt 50, Primer GC% Max 60.

In the second approach, a computational pipeline (UniAmP) composed of wrapper scripts was developed to design strain-specific primers for bacteria to use in real-time PCR. To accomplish this: (1) unique sequences to a reference genome were determined and (2) these unique sequences were then used for primer design. To find unique sequences in a reference genome, query genomes with a high sequence similarity to the reference genome were retrieved. The Genome Taxonomy Database Toolkit (GTDB-tk) v1.7.0 was used to retrieve closely related query genomes from the Genome Taxonomy Database (GTDB) (release 202) [115]. Additionally, the GenBank and RefSeq databases from the National Center for Biotechnology Information (NCBI) were remotely searched using the datasets v10.25.0 command line tool (https://github.com/ncbi/datasets). For this search, all genomes pertaining to the same genus as the reference genome were downloaded. RNAmmer v1.2 [116] was then used to extract the 16S rRNA gene sequences from these genomes. Only genomes whose 16S rRNA gene was >97% identical to the 16S rRNA gene from the reference genome were used as queries. Second, pairwise genome alignments were performed between each query genome and the reference genome using nucmer v3.1 [92]. Unique sequences in the reference genome, not found in any of the query genomes, were extracted using BedTools v2.25.0 [117]. Only unique reference genome sequences that were 150–250 bp long and contained GC content of 40–60% were selected for further processing. As one last step to confirm sequences were unique to the reference genome, pairwise local alignments were performed between each unique sequence and query sequences from the same genus in the GenBank nucleotide database. Query sequences, from the same genus, were retrieved using the e-utilities from NCBI and compared using BLASTN v2.10.0+ [111]. Only the most unique reference sequence was used for primer design. To design primers, the unique reference sequence was used in a Primer-BLAST [118] search using the specified parameters: PCR product size Min 100, PCR product size Max 200, # of primers to return 500, Database nr, Organism bacteria (taxid: 2), Primer must have at least 5 total mismatches to unintended targets, including at least 2 mismatches within the last 3 bps at the 3′ end, Primer Size Min 18, Primer Size Opt 22, Primer Size Max 26, Primer GC content (%) Min 40, Primer GC content (%) Max 60. Primer-BLAST results were saved as a HTML file and parsed using a custom Python script. In silico PCR was then performed using USEARCH v11.0.667 [119] to determine the number of amplicons in the reference genome and in a selected set of query genomes. For each bacterial strain, primers with the fewest number of non-target amplicons found in the Primer-BLAST search, only 1 reference amplicon generated from in silico PCR, and the lowest primer pair complementarity based on Primer-BLAST results were used for real-time PCR experiments. Primers were also subjected to PCR suitability tests using the PCR Primer Stats function of the online Sequence Manipulation Suite (https://www.bioinformatics.org/sms2/index.html) [120].

Strain-specific PCR reactions were prepared in a total volume of 25 μL consisting of 1× PCR buffer with Mg^2+^ (1.5 mM MgCl_2_, 10 mM KCl, 8 mM (NH_4_)_2_SO_4_, 10 mM Tris-HCl, pH 9.0, 0.05% NP-40; Thomas Scientific, Swedesboro, NJ, USA), 0.2 mM dNTPs, 0.4 µM forward primer, 0.4 µM reverse primer, 2 units of ChoiceTaq DNA polymerase (Thomas Scientific, Swedesboro, NJ, USA; catalog # CB4050-2), and 2 μL of either nucleic acids from inoculated duckweed (~100 ng/µL) or bacteria DNA (5 ng/μL). For Sp7 and DAB 1A-specific PCR, 2% and 10% DMSO were added, respectively, to end-point PCR reactions to avoid non-specific amplification due to high genome GC content (File S1). PCR reactions were executed using the following 3-stage thermocycler program: (1) a denaturation stage of 95 °C for 5 min; (2) 30 cycles consisting of 95 °C for 15 s, 60 °C for 15 s, and 72 °C for 30 s; and (3) a final extension stage of 72 °C for 5 min. Strain-specific PCR products were visualized on a 1.0% (*w/v*) agarose gel stained with ethidium bromide.

### 5.9. Quantification of Bacteria Colonization Levels on Duckweed

The colonization levels of bacteria associated with duckweed were determined by real-time PCR (qPCR) using bacterial strain-specific and *qLFY* primers (File S2). Bacterial strain-specific primers were designed by the UniAmp computational pipeline, and *qLFY* primers were designed to be complementary to the single-copy, plant-specific *LEAFY* gene (File S3). For each inoculated Lm5576 sample, bacterial strain-specific primers were used to determine bacteria DNA copy number and *qLFY* primers were used to determine Lm5576 DNA copy number. To calculate bacterial colonization levels, bacteria DNA copy number was divided by Lm5576 DNA copy number for each inoculated Lm5576 sample [48].

For each qPCR reaction, a total volume of 20 μL was used and consisted of: 250 nM forward primer, 250 nM reverse primer, 1× Power SYBR Green PCR Master Mix (Thermo Fisher Scientific, Waltham, MA, USA; catalog # 4367659), and 5 μL of either nucleic acids from inoculated duckweed (~100 ng/µL) or 5 μL of DNA from Lm5576 or bacteria alone. qPCR reactions were executed and analyzed using the StepOnePlus Real-Time PCR System from Applied Biosystems with StepOne software v2.2.2 (ThermoFisher Scientific, Waltham, MA, USA). The following settings were used: standard curve experiment; method with a holding stage of 10 min at 95 °C and cycling stage of 40 cycles consisting of 95 °C for 15 s and 60 °C for 1 min.

To determine the DNA copy number in bacteria and Lm5576, standard curves were generated using bacteria and Lm5576 DNA [48]. DNA was isolated using the nucleic acid protocol described in this study with an additional RNase treatment at 37 °C for 30–60 min prior to the chloroform washes. DNA was quantified with a NanoDrop microvolume spectrophotometer (ThermoFisher Scientific, Waltham, MA, USA). Tenfold serial dilutions were performed to generate the following DNA standards: 5, 0.5, 0.05, 0.005, 0.0005 ng/μL for bacteria DNA and 50, 5, 0.5, 0.05, 0.005 ng/μL for Lm5576 DNA. The DNA copy number was then determined for each standard using the following equation:

$$DNA\;copy\;number=\frac{DNA\;quantity\;\left(\mathrm{ng}\right)\times Avogrado^{\prime }s\;constant}{Genome\;size\;\left(\mathrm{bp}\right)\times 10^{9}\times MW\;of\;DNA}$$

where *Avogadro’s constant* is 6.022 × 10^23^, and the *MW of DNA* is 660 Da per 1 bp of double-stranded DNA. The following genome sizes were used: 6.07 Mbp for G2-6 (File S1), 3.39 Mbp for DAB 1A (File S1), 7.03 Mbp for Sp7 (JGI IMG genome id 2597490356), 7.53 Mbp for Sp245 (GenBank GCA_000237365.1), and 398.93 Mbp for Lm5576 (unpublished genome draft). The copy number of the DNA standards ranged from 10^5^ to 10^1^ copies for G2-6, Sp7, Sp245, and Lm5576, whereas a range of 10^6^ to 10^2^ copies was produced for DAB 1A. qPCR reactions were then executed for DNA standards to generate standard curves of bacteria and Lm5576 DNA copy numbers. These standards curves were then used by the StepOne software to calculate the bacteria and Lm5576 DNA copy number for inoculated Lm5576 samples. All standard curves contained an R^2^ > 0.97 and efficiencies that ranged between 82–106%. Reactions with a Ct > 30 were not considered to be positive for amplification. Bacterial strain-specific primers did not amplify Lm5576 DNA, and *qLFY* primers did not amplify any bacteria DNA. No amplification was observed in any no template controls.

## Data Availability

Raw experimental data, bioinformatic analyses, and protocols used in this study can be found on figshare (https://figshare.com/projects/Acosta_2023_Plants/155327). Protocols can also be found on figshare (https://figshare.com/projects/duckweed_microbiome/155330) and protocols.io (https://www.protocols.io/workspaces/duckweed_microbiome). The UniAmp pipeline is available at: https://github.com/kenscripts/UniAmp. Identifiers for 16S rRNA gene sequences and genomes generated in this study can be found in File S1. For the *Azospirillum brasilense* Sp7 genome, the JGI assembly with IMG genome id 2597490356 was used. For the *Azospirillum baldaniorum* Sp245 genome, the GenBank assembly GCA_000237365.1 was used.

## Supplementary Materials

The following supporting information can be downloaded at: https://www.mdpi.com/article/10.3390/plants12040872/s1. Figure S1. Nucleic acid isolation between mortar & pestle and bead-beating. Figure S2. Nucleic acid isolation from Lm5576 with bead-beating. Figure S3. Nucleic acid isolation from bacteria with bead-beating. Figure S4. Optimization of nucleic acid extraction using a bead-beating protocol. Figure S5. Nucleic acid isolation from different bacteria. Figure S6. Amplification of bacteria DNA using RISA primers. Figure S7. Optimization of *LEAFY* gene PCR. Figure S8. Overview of UniAmp computational pipeline to design strain-specific primers. File S1. Metadata of bacterial isolates used in this study. File S2. Information on primers used in this study. File S3. Design of duckweed *LEAFY* gene primers. File S4. Strain-specific primers generated using UniAmp computational pipeline. File S5. Three-dimensional confocal microscopy of inoculated Lm5576 samples. File S6. Attachment PCR results of confocal microscopy samples.
