# Supplementary material for: Optimization of Molecular Methods for Detecting Duckweed-Associated Bacteria
Source: Plants (Basel). 2023 Feb 15;12(4):872. doi: 10.3390/plants12040872 (PMC9965182; doi:10.3390/plants12040872)
Supplement: Supplementary file 1 [file plants-12-00872-s001.zip › Supplemental files for Acosta et al. Plants'23_final2/FileS6.pptx]

## Slide 1
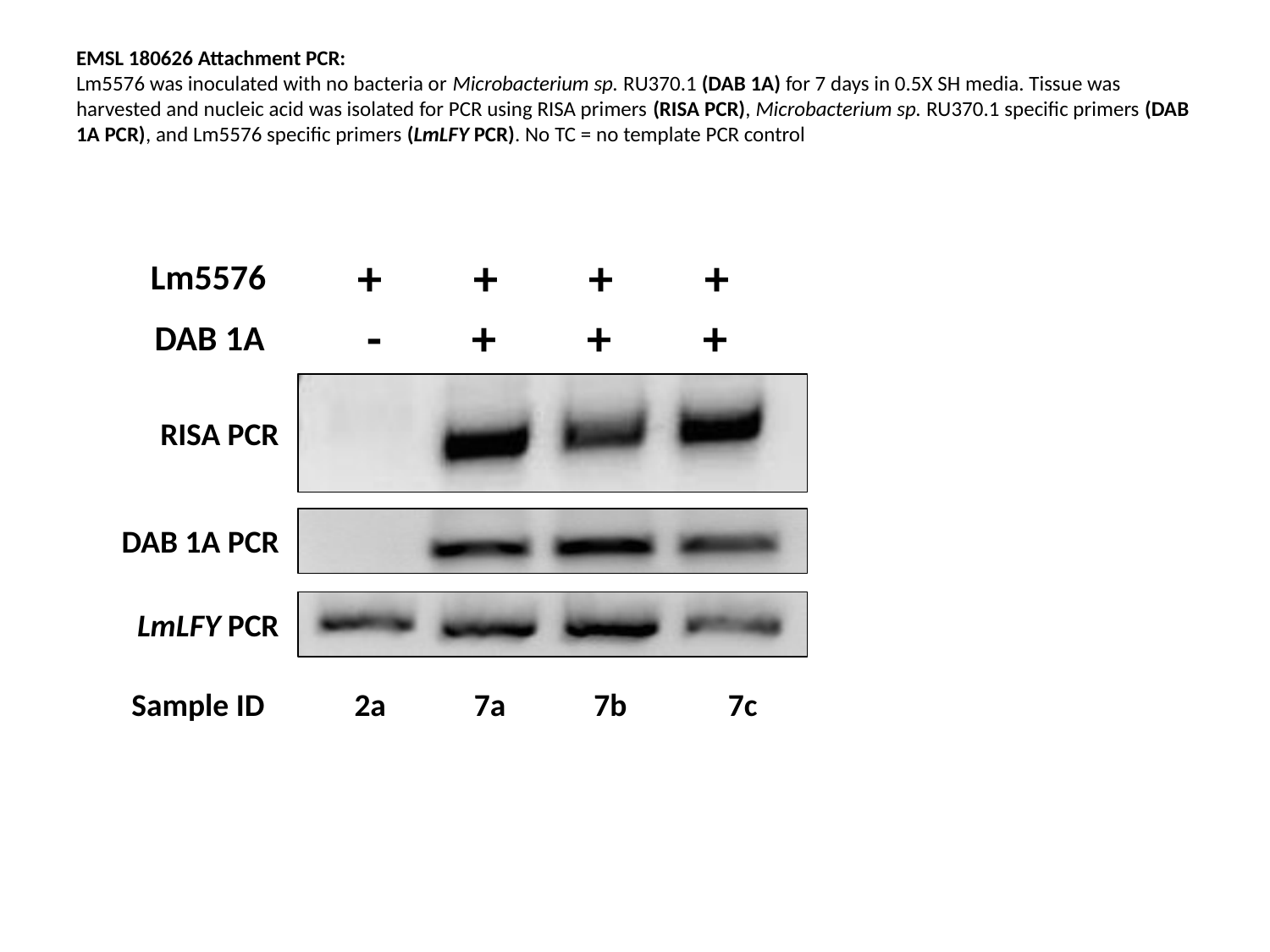

# EMSL 180626 Attachment PCR: Lm5576 was inoculated with no bacteria or Microbacterium sp. RU370.1 (DAB 1A) for 7 days in 0.5X SH media. Tissue was harvested and nucleic acid was isolated for PCR using RISA primers (RISA PCR), Microbacterium sp. RU370.1 specific primers (DAB 1A PCR), and Lm5576 specific primers (LmLFY PCR). No TC = no template PCR control
 +	 + 	 +	 +
Lm5576
 -	 + 	 +	 +
DAB 1A
RISA PCR
DAB 1A PCR
LmLFY PCR
Sample ID
 2a	 7a	 7b	 7c

## Slide 2
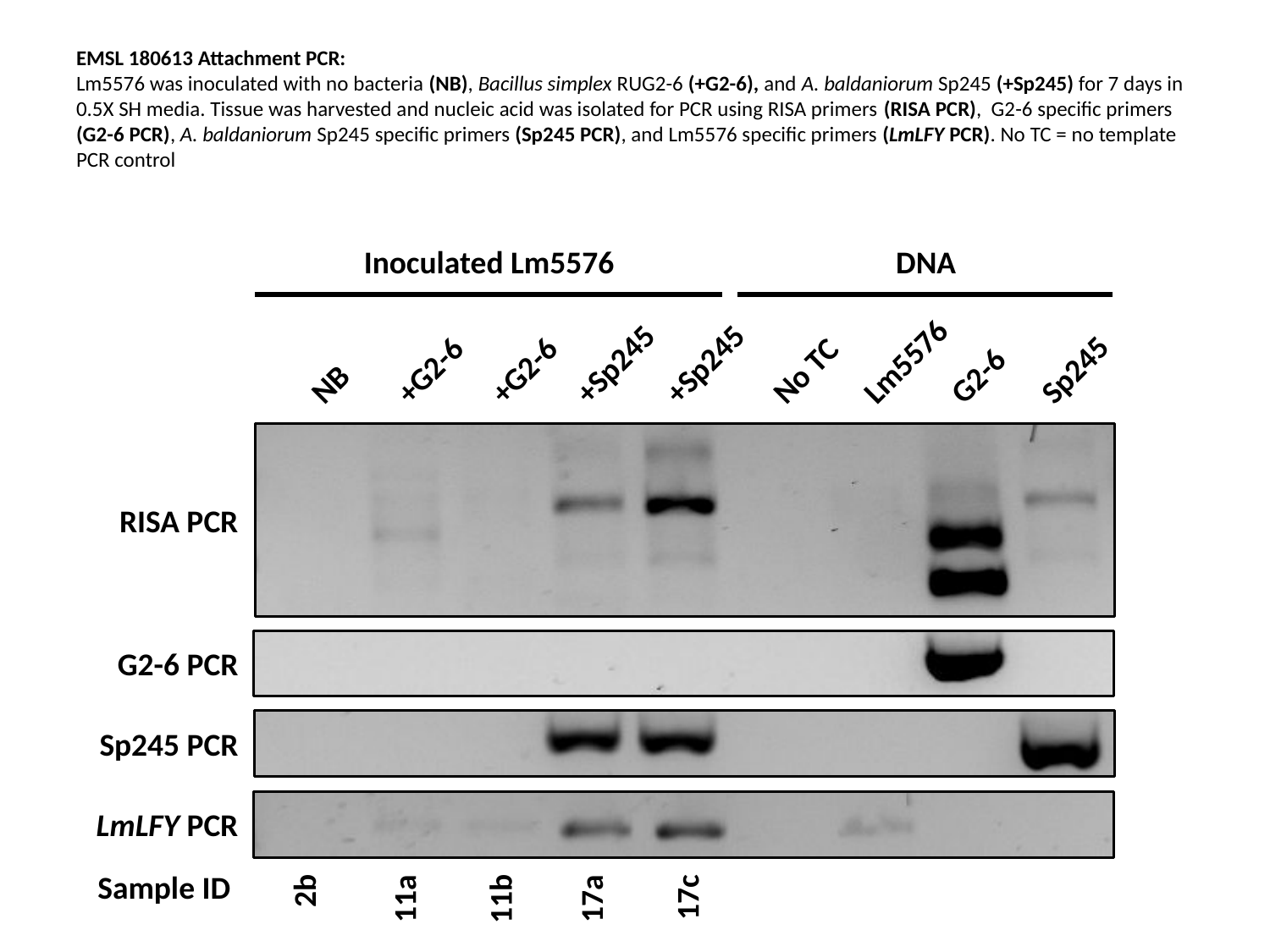

# EMSL 180613 Attachment PCR: Lm5576 was inoculated with no bacteria (NB), Bacillus simplex RUG2-6 (+G2-6), and A. baldaniorum Sp245 (+Sp245) for 7 days in 0.5X SH media. Tissue was harvested and nucleic acid was isolated for PCR using RISA primers (RISA PCR), G2-6 specific primers (G2-6 PCR), A. baldaniorum Sp245 specific primers (Sp245 PCR), and Lm5576 specific primers (LmLFY PCR). No TC = no template PCR control
Inoculated Lm5576
DNA
Sp245
G2-6
Lm5576
+Sp245
+Sp245
+G2-6
+G2-6
No TC
NB
RISA PCR
G2-6 PCR
Sp245 PCR
LmLFY PCR
Sample ID
2b
17c
11a
17a
11b

## Slide 3
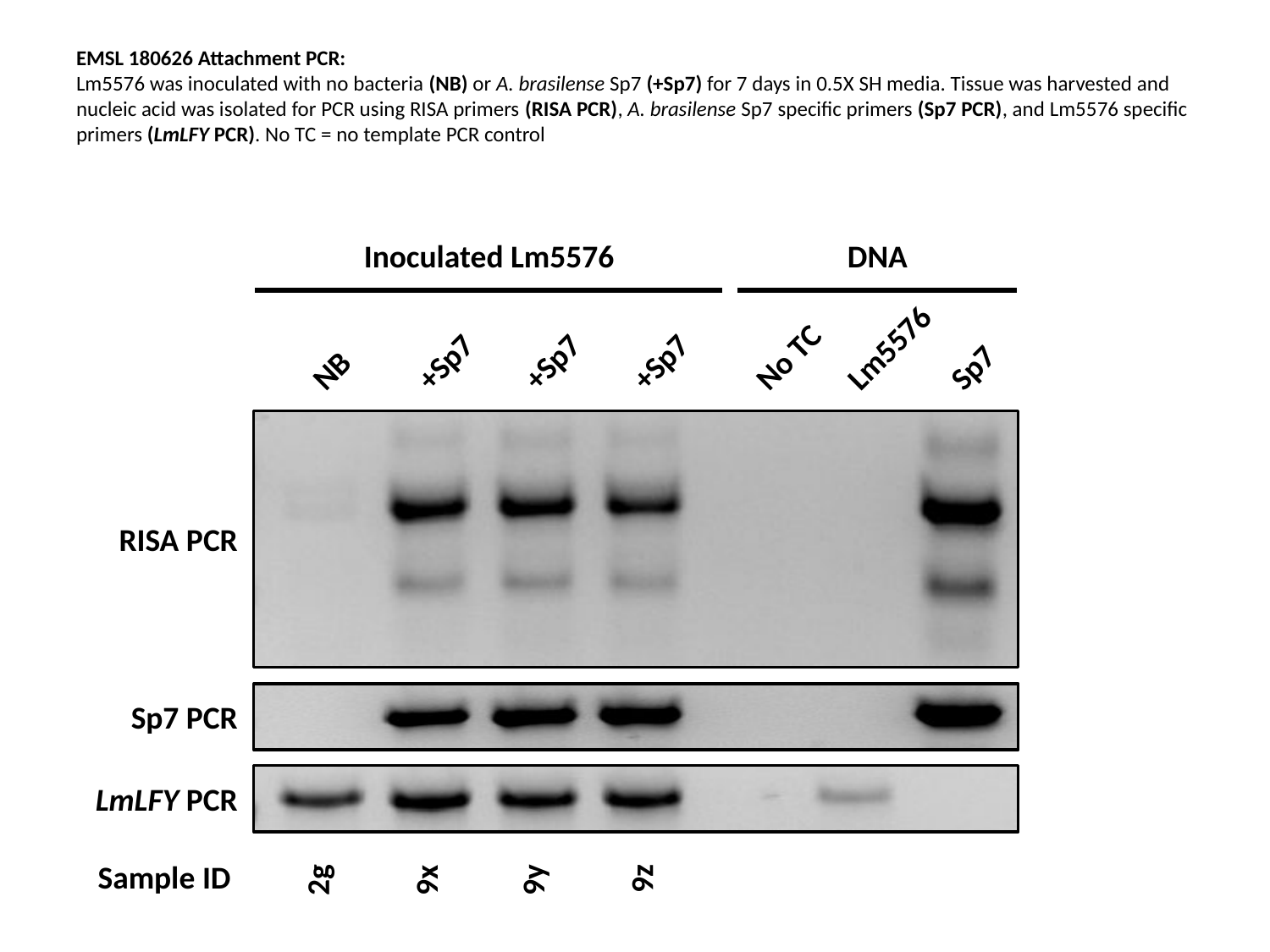

# EMSL 180626 Attachment PCR: Lm5576 was inoculated with no bacteria (NB) or A. brasilense Sp7 (+Sp7) for 7 days in 0.5X SH media. Tissue was harvested and nucleic acid was isolated for PCR using RISA primers (RISA PCR), A. brasilense Sp7 specific primers (Sp7 PCR), and Lm5576 specific primers (LmLFY PCR). No TC = no template PCR control
Inoculated Lm5576
DNA
Sp7
Lm5576
No TC
+Sp7
+Sp7
+Sp7
NB
RISA PCR
Sp7 PCR
LmLFY PCR
Sample ID
9z
2g
9x
9y
