# Supplementary material for: Optimization of Molecular Methods for Detecting Duckweed-Associated Bacteria
Source: Plants (Basel). 2023 Feb 15;12(4):872. doi: 10.3390/plants12040872 (PMC9965182; doi:10.3390/plants12040872)
Supplement: Supplementary file 1 [file plants-12-00872-s001.zip › Supplemental files for Acosta et al. Plants'23_final2/Supplementary Material Information_final.docx]

**Supplementary Material Information**

**Figure S1. Nucleic acid isolation between mortar & pestle and bead-beating.** Nucleic acid extraction was compared between mortar & pestle (M&P) and bead-beating (BB), using CTAB as the lysis buffer. **A)** Total micrograms (ug) of nucleic acids extracted per 10 plants of Lm5576 using bead-beating or mortar & pestle. To calculate the total ug of nucleic acid extracted, the nucleic acid concentration of the extract was multiplied by the total extract volume. **B)** Gel electrophoresis of approximately 500 nanograms of Lm5576 nucleic acids isolated with bead-beating or using mortar & pestle. **C)** Concentration of nucleic acids extracted from bacteria using bead-beating or mortar & pestle. The total micrograms (ug) of nucleic acid isolated was calculated by multiplying the nucleic acid concentration of extracts by the total extract volume. The total ug of nucleic acids isolated was then normalized to the optical density at 600 nm (OD_600_) of the liquid bacterial culture used for extraction. 1A = nucleic acids isolated from *Microbacterium sp.* RU370.1 (DAB 1A); 3D = nucleic acids isolated from *Bacillus sp.* RU9509.4 (DAB 3D). **D)** Gel electrophoresis of approximately 500 nanograms of bacterial nucleic acids isolated with bead-beating or using mortar & pestle.

**Figure S2. Nucleic acid isolation from Lm5576 with bead-beating.** Different-sized beads were tested for their efficacy to extract nucleic acid from Lm5576. **A)** Homogenization of Lm5576 tissue by different bead sizes. **B)** Gel electrophoresis of approximately 500 nanograms of nucleic acid isolated from Lm5576 using different bead sizes. M = Mixed; 0.1 = 1 gram of 100 um silica beads; 1.7 = 1 gram of 1.7 mm zirconium beads; 4 = (1) 4 mm glass bead; Mixed = 0.5 grams of 100 um silica beads, 0.5 grams of 1.7 zirconium beads, and (1) 4 mm glass bead; Lm5576 = *Lemna minor* 5576. **C)** Total micrograms (ug) of nucleic acids extracted per 10 plants of Lm5576 using different bead sizes. To calculate the total ug of nucleic acid extracted, the nucleic acid concentration of the extract was multiplied by the total extract volume.

**Figure S3. Nucleic acid isolation from bacteria with bead-beating.** Different-sized beads were tested for extracting nucleic acid from bacteria. **A)** Gel electrophoresis of approximately 500 nanograms of nucleic acid isolated from bacteria using different bead sizes. M = Mixed; 0.1 = 1 gram of 100 um silica beads; 1.7 = 1 gram of 1.7 mm zirconium beads; 4 = (1) 4 mm glass bead; Mixed = 0.5 grams of 100 um beads, 0.5 grams of 1.7 zirconium beads, and (1) 4 mm glass bead; DAB 37A = nucleic acids isolated from DAB isolate 37A; Sp245 = nucleic acids isolated from *Azospirillum baldaniorum* Sp245. **B)** Concentration of nucleic acids extracted from bacteria using different bead sizes. The total micrograms (ug) of nucleic acid isolated was calculated by multiplying the nucleic acid concentration of extracts by the total extract volume. The total ug of nucleic acids isolated was then normalized to the optical density at 600 nm (OD_600_) of the liquid bacterial culture used for extraction.

**Figure S4. Optimization of nucleic acid extraction using a bead-beating protocol.** Modifications to the lysis step of the bead-beating protocol were tested to improve nucleic acid extraction. **A)** Total micrograms (ug) of nucleic acids extracted per 10 plants of Lm5576 using different lysis modifications. To calculate the total ug of nucleic acid extracted, the nucleic acid concentration of the extract was multiplied by the total extract volume. CTAB = 600 uL CTAB lysis buffer; CTAB+Chloroform = 300 uL CTAB and 300 uL chloroform lysis buffer; CTAB+65^o^C Heating Step = 600 uL CTAB lysis buffer with 65^o^C heating step after lysis.

**B)** Gel electrophoresis of approximately 500 nanograms of nucleic acid isolated from bacteria using different lysis conditions. 4 = CTAB/chloroform lysis buffer and bead-beating at 4^o^C; 4+ = CTAB/chloroform lysis buffer plus 25 uL beta-mercaptoethanol and bead-beating at 4^o^C; RT = CTAB/chloroform lysis buffer and bead-beating at room temperature; RT+ = CTAB/chloroform lysis buffer plus 25 uL beta-mercaptoethanol and bead-beating at room temperature; Sp245 = nucleic acids isolated from *Azospirillum baldaniorum* Sp245; G2-6 = nucleic acids isolated from *Bacillus simplex* RUG2-6. **C)** Concentration of nucleic acids extracted from bacteria using different lysis conditions. The total micrograms (ug) of nucleic acid isolated was calculated by multiplying the nucleic acid concentration of extracts by the total extract volume. The total ug of nucleic acids isolated was then normalized to the optical density at 600 nm (OD_600_) of the liquid bacterial culture used for extraction.

**Figure S5. Nucleic acid isolation from different bacteria.** Nucleic acids from different bacteria were extracted using the bead-beating protocol with different incubation times in the CTAB/chloroform lysis buffer. **A)** Gel electrophoresis of approximately 500 nanograms of nucleic acids isolated from different bacteria using different incubation times in lysis buffer. 0 = no incubation in lysis buffer; 15 = 15-minute incubation in lysis buffer; 30 = 30-minute incubation in lysis buffer; 60 = 60-minute incubation in lysis buffer; G2-6 = nucleic acids isolated from *Bacillus simplex* RUG2-6; DAB 1A = nucleic acids isolated from *Microbacterium sp.* RU370.1; DAB 3D = nucleic acids isolated from *Bacillus sp.* RU9509.4; Sp7 = nucleic acids isolated from *Azospirillum brasilense* Sp7; Sp245 = nucleic acids isolated from *Azospirillum baldaniorum* Sp245. **B)** Concentration of nucleic acids extracted from bacteria using different incubation times in lysis buffer. The total micrograms (ug) of nucleic acid isolated was calculated by multiplying the nucleic acid concentration of extracts by the total extract volume. The total ug of nucleic acids isolated was then normalized to the optical density at 600 nm (OD_600_) of the liquid bacterial culture used for extraction.

**Figure S6. Amplification of bacteria DNA using RISA primers. A)** Addition of magnesium chloride improves the amplification of bacteria DNA using RISA primers. Buffer = Choice Taq polymerase buffer (already contains 1.5 mM MgCl_2_); A = 16S-e1390f and 23S-e130r; B = 16S-e1390f and 23S-e205r; C = 16S-e1390f and 23S-e474r **B)** Different RISA primers were tested for their ability to amplify duckweed DNA. Lm5576-S = sterile *Lemna minor* 5576; dw9509-S = sterile *Spirodela polyrhiza* 9509 **C)** RISA primers, 16S-e1390f and 23S-e130r, produce distinct fingerprints for different bacteria. NTC = no template control; *E. coli* = *Escherichia coli*; DAB 1A = *Microbacterium sp.* RU370.1; DAB 3D = *Bacillus sp.* RU9509.4; *A.tumefaciens* = *Agrobacterium tumefaciens*.

**Figure S7. Optimization of *LEAFY* gene PCR. A)** *LEAFY* gene PCR was performed on nucleic acids from bacteria and Lm5576 at different annealing temperatures. No TC = no template control; Sp7 = *Azospirillum brasilense* Sp7; Lm5576 = *Lemna minor* 5576 **B)** *LEAFY* gene PCR of Lm5576 and dw9509 nucleic acid at different concentrations using a different number of PCR cycles. * = the number of cycles selected for *LEAFY* gene PCR; *LmLFY* = PCR using LmLFY-F and LmLFY-R primers to amplify *LEAFY* gene from Lm5576; *SpLFY* = PCR using SpLFY-F and SpLFY-R primers to amplify *LEAFY* gene from dw5909.

**Figure S8. Overview of UniAmp computational pipeline to design strain-specific primers.** The UniAmp pipeline can be conceptually split into 4 modules: 1) build a directory of query genomes, 2) retrieve unique sequences in a reference genome compared to query genomes, 3) select a unique reference sequence for primer design, and 4) design primers to the unique reference sequence.

**File S1. Metadata of bacterial isolates used in this study. A)** Isolation details, taxonomy, and colony morphology of bacterial isolates used in this study. Consensus 16S rRNA gene sequences were annotated with RDP classifier v.2.13 using the 16S rRNA training set 18. **B)** Information on genomes generated in this study.

**File S2. Information on primers used in this study.**

**File S3. Design of duckweed *LEAFY* gene primers.** Pairwise global alignment of *LEAFY* genes from *L. minor* 5500 and *S. polyrhiza* 9509. qLFY-F and qLFY-R = *LEAFY* gene primers used in real-time PCR to quantify *L. minor* and *S. polyrhiza* DNA; SpLFY-F and SpLFY-R = *LEAFY* gene primers specific to *S. polyrhiza* and used in end-point PCR; LmLFY-F and LmLFY-R = *LEAFY* gene primers specific to *L. minor* and used in end-point PCR.

**File S4. Strain-specific primers generated using UniAmp computational pipeline.** Primer pairs highlighted in yellow were used in this study to detect the colonization of Lm5576 by G2-6, DAB 1A, Sp7, and Sp245 bacteria. Nontargets_organisms = number of non-targets amplified determined by Primer-BLAST, Organisms_amplified = number of organisms amplified determined by Primer-BLAST, For_pr_seq = forward primer sequence, Rev_pr_seq = reverse primer sequence, Self_complementarity = determined by Primer-BLAST, Self_3’_complementarity = determined by Primer-BLAST, Total_prpair_complementarity = sum of Self_complementarity and Self_3’_complementarity, Ref_amplicons = number of amplicons found in reference genome by UniAmp, Nonref_amplicons = number of amplicons found in selected query genomes by UniAmp, SMS_notes = manually curated notes from Sequence Manipulation Suite results.

**File S5. Three-dimensional confocal microscopy of inoculated Lm5576 samples.** Calcofluor white was used to stain plant cellulose and visualized with the blue channel, SYBR Gold was used to stain DNA and visualized with the green channel, and chlorophyll autofluorescence was visualized with the red channel. Both bacterial and Lm5576 DNA were stained by SYBR Gold and pictured in green, but bacterial DNA (indicated by white arrows) is smaller in size than Lm5576 nuclei (indicated by red arrows), and these bacteria are often found in clustered colonies.

**File S6. Attachment PCR results of confocal microscopy samples.**
