# Supplementary material for: Optimization of Molecular Methods for Detecting Duckweed-Associated Bacteria
Source: Plants (Basel). 2023 Feb 15;12(4):872. doi: 10.3390/plants12040872 (PMC9965182; doi:10.3390/plants12040872)

**MUSCLE (3.8) multiple sequence alignment**

**Sp17g00070 ATG---CCCACGCAAGCGGATACACTTCGGAGTGACGCATTGTGCAGAGCCGCCGCAGTT**

**Lminor_018706 ATGGATCCGATGGAAGCG-------TTCAGA-TGCTGGGACATG-AGAATGGCGGCGCTG**

***** ** * * ***** *** ** ** * ** *** ** ** ***

**Sp17g00070 TCCACAGCCTCCACGGCGGCGGAGGGCGAGAAACCGAGAGAGGCAGGTTCTAAGAGGGAC**

**Lminor_018706 CTAGCAACCCAAGTGGCGGCG------------CCGCTGGATGCGG--------------**

**** ** ******* *** ** ** ***

**Sp17g00070 GAAGGAAACAAAAAGGCCTGCGGCAGCCGCCTAACAAATAATTCAATAATTTTAAATAAA**

**Lminor_018706 ------------------CGGCACAGCCGCC-----------------------------**

*** **********

**Sp17g00070 ATACCTGACAGTTTTATATCTAATAATGGCATGAAAAAATACGGTATAAGTATTTTTTCA**

**Lminor_018706 ------------------------------------------------------------**

**Sp17g00070 CTGATACTTAAAATTTAAAAAATATTAAAAAAATAATCCATTATATTTATTTTGAAAATT**

**Lminor_018706 ------------------------------------------------------------**

**Sp17g00070 AAATAATTCTTGTGTGGTATACTGAATAAGAGTACATTGGGAATAGGCAGCATATGGGCA**

**Lminor_018706 ------------------------------------------------------------**

**Sp17g00070 ACGCCTTCTAAACACCAAGGAAGAGCGAGGATGGATATTAAATAATTTTTAATTGAAAAT**

**Lminor_018706 ------------------------GCGAGGA-----------------------------**

***********

**Sp17g00070 TAATGTGTATGTCAAAAATATCAAACCCAAGTGGTGCACAAAATGTGGAACACCAAATTG**

**Lminor_018706 --------------------------------GGCCTGCAAG------------------**

**** *****

**Sp17g00070 TATCGATGCTCATATGTTATTATATGTTCCAAAATTGCATAATATATCAAAATTGGTAAA**

**Lminor_018706 ---------------------AGATGTTCCAGGATTACGGGGTG----------------**

*** ******** *** * ***

**Sp17g00070 TCAATATATTGCTAGTTACATTGCTGAACAACTGAGTATATCAATGTTAAAATGTCAATA**

**Lminor_018706 ------------------------------------------------------------**

**Sp17g00070 TGCCATGCATCTAAATATCAAAGTATATCAATGTCAATATTTAAAAATATACATTTTAAG**

**Lminor_018706 ------------AGATATCAGA--------------------------------------**

*** ****** ***

**Sp17g00070 TTGCAAAAATATAAGGACTAACCTTGAGAGTAGTATGCAAATAATTTTAATATAAAATTC**

**Lminor_018706 ------------------------------------------------------------**

**Sp17g00070 AATATTTCAGTATATAAAGTTTGATCTACGACATAACATGATATATCAATGTGAAAATAT**

**Lminor_018706 ----------------------------------------------CGGTGGGGAA----**

*** ** * ****

**Sp17g00070 TAACATATGACGACTTACATTATGGAGCAGCAGTGATTGATATGTTAGCGTGTGAAGAGT**

**Lminor_018706 --------------------------------------GAT-----------CGGAGAGT**

***** * *******

**Sp17g00070 TGGGTAGCAAAAGAATAATCTATATAAAGGTTAAAATGCTAGTATACAATGAGTGAGTTA**

**Lminor_018706 TGGG-------------------------------------GTTTACGGTGAGCACGCTA**

****** ** *** **** * ****

**Sp17g00070 TATTTGTAGAATTATATATTGTATAAAGCCTAATATCTTTGGACATGAAAAGCATATTGT**

**Lminor_018706 ------------------------------------------------------------**

**Sp17g00070 GAAATATAAATATCGAACTCAACAGTAGTACATGAATAATGACATATATTTAACATAATA**

**Lminor_018706 ------------------------------------------------------------**

**Sp17g00070 TTTTAGAGTATGAAATAATTTTTACAATATAGTGTATAAAGAACAATATATGTATTTATG**

**Lminor_018706 ------------------------------------------------------------**

**Sp17g00070 ATTTGATATAGGCTAGTATGTAAAGTTAAATATCTCAAACATTAAATGTTTGTGATGACA**

**Lminor_018706 ---------------------------------------------------GTGGCGATG**

***** ****

**Sp17g00070 AAACTTAATTTGATATTATTTTTTATTCATACATAGTTGGATGAATTGGGAGAGGGGTTG**

**Lminor_018706 AA------------------------------------GGAGGAAGAAGTAGAGG-----**

**** *** *** * *******

**Sp17g00070 AATTCTAAATTTTTTTATAATTAAATATATACTTTTATTTTATTTAAGTTAAATTTATTT**

**Lminor_018706 ------------------------------------------------------------**

**Sp17g00070 ATAATTATATATATGAACACAAGTTTTTATAGTTGTTTGGCAAAAAAAAAAAATTAACAT**

**Lminor_018706 ----------ACATG----------CTTGCAGCTCTGTGCC-------------------**

*** *** ** ** * * ** ***

**Sp17g00070 ATGTTTTCATATGAATTCACCTTGGTTCACTATTATAGTATTGAAATGTACATGACTTTC**

**Lminor_018706 ATGTTTTC----------------------------------------------------**

************

**Sp17g00070 TAAGATTGGAATTCCTCACACTATTAAACTATATGAGTTTATGATCACTCCACTTAAAAT**

**Lminor_018706 --CGGTGGGAGCTTCTC-------------GTCGGAG-----------------------**

*** * *** * *** * *****

**Sp17g00070 CTTAAGGATTAATAGCTCACAAATCTCCTGTATTATAGATACACCATAGGTGGCTCAATA**

**Lminor_018706 ----------------------------------AGAGATACGGCATAA-----------**

*** ****** ******

**Sp17g00070 ACCTATTTAATTGTATTGCCAACACACCACAAGTGCTTCAAAGAGTCCAGATGTCATCAC**

**Lminor_018706 --------------------AAGCCGCCATAAGGAC------------------------**

**** * *** *** ***

**Sp17g00070 TCACACAAGAATAATATATCAATTTTGGATTGATGTTGCTATTCCCTTCAGCTTTGATTA**

**Lminor_018706 --------------------------GGAGCGGCGCCGCC--------------------**

***** * * ****

**Sp17g00070 TTGTTTGATTTAATGTGAGTTGTTGAAGATTTTTACAAGAGCCAGGTTGAATGTTCTTTG**

**Lminor_018706 ----------------------------------------GCCAGCTTGATGGGTTTTT-**

******* **** * * *****

**Sp17g00070 AAATTGATATTCATTTTCATGTGCATAGCTCCCTTAAAAAATCTCAATCTTGCATATGAA**

**Lminor_018706 -----------------------------TCCC---------------------------**

********

**Sp17g00070 TCTTTTAGAACTTGAAAATAGGTGGTCGAGAGGAGAAAGCCCTCAAAGCAACCGCAACAA**

**Lminor_018706 --------------------------CCGGAGGAGGAGG-------AGCAGCGGCGGCGG**

*** ****** * * **** * ** ***

**Sp17g00070 TCTAAATTTTCATTTGAATTTTAGTGCCCTAAATTTGGACAACATGGTTCTTTGATAGTA**

**Lminor_018706 CGCCAGTTTCCCT-------------------GCTCGGACGCC-----------------**

*** *** * * * **** ***

**Sp17g00070 AAATATAGAGGGCCGGCCTGCCCATTTTTTTTATTGGATATAATGACCAACTTTGGTGGA**

**Lminor_018706 -----------GCCG------CCAT-----------------------------------**

****** ******

**Sp17g00070 CCTTAGTGGGCTGCACATATGTTTTGAAAAACACAAACTTATATTCATAAAACTTATAAA**

**Lminor_018706 --------GGATGCGC--------------------------------------------**

**** *** ***

**Sp17g00070 AATCATAAATCTTCAAGTATTTTGAATTTTGGTGAGCAATTTTTTGTGTTAAATTTTTAT**

**Lminor_018706 ------------------------------------------------------------**

**Sp17g00070 TTGAATGATTTTTCCATTAATTGTTGTATGCTTGTATTTGTAACATATTTTATCAGATAA**

**Lminor_018706 ------------------------------------------------------------**

**Sp17g00070 AAATTATTCTAGTTATATAGGTTAAACTTCTTTATTATTTTATCCTTAGGTAGAAATATA**

**Lminor_018706 ------------------------------------------------------------**

**Sp17g00070 ATTTTATTAATCTTGCAAAACTAATATCAATTCATTACATTTGATAACTTCTTACAAAAC**

**Lminor_018706 ----------TCTCGCAA------------------------------------------**

***** ******

**Sp17g00070 TAGTTGCATTTGACTCCTCACTTGAAGAGCTGTTCAATACCACGTGTCTATGTCAATGAA**

**Lminor_018706 -----------------------GAAGGGC------------------------------**

****** ****

**Sp17g00070 GCTACGTGTTACAAAAACTTAAAAATATTAGGACACATGCATAGGCTAATAATCATTTTA**

**Lminor_018706 ------------------------------------------------------------**

**Sp17g00070 CCTTGCACTAACTTCTTATTACATTTATAAGCAAAAAGTTTAGCTTTTATCCTCCTAATG**

**Lminor_018706 ------------------------------------------------------------**

**Sp17g00070 GGAATCAAATGGTAAGTGAGACTTGTTTAATATTTAACAATTAATATCATCAACCAAAAC**

**Lminor_018706 ------------------------------------------------------------**

**Sp17g00070 ATTTTATTAATTAAAATAAATGAAATTTGAATCATAATTTCACATTAAATCTCATCAATG**

**Lminor_018706 ------------------------------------------------------------**

**Sp17g00070 TCATAATTCCGAATCATGCATGACTTTACTAAATTTTCTTGCTAAATAATTAAAACACTA**

**Lminor_018706 ------------------------------------------------------------**

**Sp17g00070 TTGGGGTAATCTATAAATGCAATTCTCTCTCTCTTTTATGATTATAATATCATGAAATTG**

**Lminor_018706 ------------------------------------------------------------**

**Sp17g00070 AACTTTTTTTTTCAAATTCATCCCTCAATTCATATCTCACTTTTCTCTATTCTCTCCAAT**

**Lminor_018706 ------------------------------------------------------------**

**Sp17g00070 TCAATATCATAAATATTGAACTTAAAATTAGTTTTTCAGTGGACAAATCATACCTCAATT**

**Lminor_018706 ------------------------------------------------------------**

**Sp17g00070 TTGCTACCAATACTAATGACAATTTTCTTCAAATAATGATTATTAAAATTTTTGATAGAA**

**Lminor_018706 -------------------------TCTCCGAAGAACGA---------------------**

***** * ** ** ****

**Sp17g00070 AACTAATGGTATGTCAAGTAAATTTCATGGTATTTTTTCAAGTGGATCATTGTATACATC**

**Lminor_018706 ------------------------------------------------------------**

**Sp17g00070 ATAATTATGACTATTTTCGAGTTAATGCAACATTATATTTCATTATTACAATTTTATGAT**

**Lminor_018706 ------------------------------------------------------------**

**Sp17g00070 ATAGATATGATGTGAAATGTGAATCAACAAGTACACATAATTTATACATATGCCCTATGA**

**Lminor_018706 ------------------------------------------------------------**

**Sp17g00070 TTTTAGGCATTGTAACTGCAATAATTTGATTTTTAGAGTAATTGCAAAAAAAATCATCTG**

**Lminor_018706 ------------------------------------------------------------**

**Sp17g00070 ATGTAATCCTTGCAAGACTATTTAATCATAAATATACTATGTATGATACAAAAATCAAAT**

**Lminor_018706 ------------------------------------------------------------**

**Sp17g00070 GTATGCTCTATATCACCTATTTTCTATTTTACTTATATTATATTTAATACTTAAATCATT**

**Lminor_018706 ------------------------------------------------------------**

**Sp17g00070 CAATACATAAATTACAACAATATGAAGGTATCTACTTTTCATCCTCTATAGTTCAGCTAA**

**Lminor_018706 -----------------------------------------------------CAGCTGG**

*********

**Sp17g00070 AGATAATTCTTTTGATACTTTGGATGTCCCATATAAAATAAATGTTTCACTAAAGGGACC**

**Lminor_018706 AGATAGTT----------------------------------------------------**

******* ****

**Sp17g00070 AAAGTTTCTCTAGGGCTGAATTTTCTATAATATATGCAATTTTAGGCATCCAAGCTCATT**

**Lminor_018706 --------TCAAGTGCTG------------------------------------------**

**** ** ******

**Sp17g00070 TTATCTTTCTCTAACTATTTCTAATTTGAGGTTATATTATAAATTTTTATTAATATGATG**

**Lminor_018706 ------------------------------------------------------------**

**Sp17g00070 GTTTGTAGATTAGATATGCAAACTTGGATAATTATTTGTTTGCGAAGTGGGTGAAAAAAT**

**Lminor_018706 -----------------------------------------GCGAGCCGTGTGAA-----**

****** * *******

**Sp17g00070 TTATTTTTTAATGAAGTCATAGTAAGAGAATTTTTATATTTTCTAAAATAATTATATGTT**

**Lminor_018706 ------------------------------------------------------------**

**Sp17g00070 TACAAGTATCATGATATCTAAATCACTTTGTTACTGTGTTTACACTAAAATTTCATCACT**

**Lminor_018706 ------------------------------------------------------------**

**Sp17g00070 TCAGCTATTTTTTCTCTCCCTAAATTTGTTTAAACAAGTTTGAATCCATAAATTTTATGT**

**Lminor_018706 ------------------------------------------------------------**

**Sp17g00070 ATTCAGGTGTGATAATTATAATTCTTGATTTTTACACATGTTCATGATCCTCGTATTTAT**

**Lminor_018706 ------------------------------------------------------------**

**Sp17g00070 CCTTAGTTTCTTGAAATTCTATTTCAATTATGCATTTTATTTCATCAAATTATTTTATAT**

**Lminor_018706 ------------------------------------------------------------**

**Sp17g00070 TTTTTATTTATAATTTTTTTTATTTATAATTTTTTTATTTATTTTTATTTATAATTTTTT**

**Lminor_018706 ------------------------------------------------------------**

**Sp17g00070 TTTATTTATGTATTGAACCTTTAAGTTTAGTATATTTTTTTTGCATGTAATCATCAATTC**

**Lminor_018706 ------------------------------------------------------------**

**Sp17g00070 TTCAAATGAATATAGAAGTATTTATTTGGATAAATTAGATTCATCAAAATAGTGTGCCTC**

**Lminor_018706 ------------------------------------------------------------**

**Sp17g00070 AATAATTTGTGTTGTAAGTTAAATTTTGGTTGTTTGGTTCAAAGAAATTATTCTTACTTG**

**Lminor_018706 ------------------------------------------------------------**

**Sp17g00070 TTGACTTTCCTTCATATGTGATCCTAAGTTTGTCCCATATTTTGTTTAATGTACTTCAAC**

**Lminor_018706 ------------------------------------------------------------**

**Sp17g00070 TTAAAGTGTAGTTAAATTCACTCATGTCAAAAGCACAATGGAGAAAATTTGCAACCTTTT**

**Lminor_018706 ------------------------------------------------------------**

**Sp17g00070 TTATATTAGATTCATTTCTATATGAGTTATATTTAACTATACTCAAAATATCAAGATCAA**

**Lminor_018706 ------------------------------------------------------------**

**Sp17g00070 AAGTTTTAATAAATAATATATATATATATATATATATATTATGTGTAATTTGTGCCAACA**

**Lminor_018706 ------------------------------------------------------------**

**Sp17g00070 AATATAAGAGGTGTTTATGTGAAATGTCCTTAGACCCATAAACATCTTGAAGTGCAAAAC**

**Lminor_018706 ---------------------------------ACCCAGAA-------GAAG--------**

******* ** ******

**Sp17g00070 CATTGGGATCTATTCTTATGGATAGTTACATCTTGTGATTTGAGCTAGCCTCAAATACCA**

**Lminor_018706 ------------------------------------------------------------**

**Sp17g00070 ACTATTGGTACCTAGATAGCCATACGAATTGATTTTAAATTAATTATAATAAACTATGCT**

**Lminor_018706 ------------------------------------------------------------**

**Sp17g00070 ATATATTATATATTAAAATATAATTTTAAATTAATATATAAAGTATAAAATAAATAAAGA**

**Lminor_018706 ------------------------------------------------------------**

**Sp17g00070 ATAATTGGTGAACATAAAGTTCTATCATAGTTTGACCTGCTTTGCACTAAATTCACTTTC**

**Lminor_018706 ------------------------------------------------------------**

**Sp17g00070 AAACAATCTACGTTGGTTCTACCACTTCATCAGCATAATATATTTGATTTTCTTAAGATA**

**Lminor_018706 ------------------------------------------------------------**

**Sp17g00070 AAAAACTCCTTACATTATTATGCTCCATGAACATGTAATCACTTTACTTAAACCTTAGGA**

**Lminor_018706 ------------------------------------------------------------**

**Sp17g00070 ATTACAATCATATATGTTTAATATTGCACGCACACCACATATGTCTCAATAATTTGGATC**

**Lminor_018706 ------------------------------------------------------------**

**Sp17g00070 ATTGCATGCCAAGGCACCACAAATGCCTCAAGTAGTTCACATATTATCATTTTATAACTC**

**Lminor_018706 ------------------------------------------------------------**

**Sp17g00070 AACAACTATTTATATGTATATGCTTATAAATCATGCATAAATTTGAGAGCAATATTAAAA**

**Lminor_018706 ------------------------------------------------------------**

**Sp17g00070 CACAGTATTAAAAAAATGATATTGTATTAATAGAATAAAGCTATTTGTTTGATATATGCT**

**Lminor_018706 ---------AAGAAAACGAT----------------------------------------**

**** **** *****

**Sp17g00070 GATTATTACTAGAATAATGTGAAATGAGACTTTCCTAACTATTGAAAAATTATCAAATAG**

**Lminor_018706 ------------------------------------------------------------**

**Sp17g00070 AGCAAAATTTATATTAGCAATGTCAATAATATGATTACAAGCTAAATATCCATCAACAAG**

**Lminor_018706 ------------------------------------------------------------**

**Sp17g00070 CTTTAAATGTGAGAAGTTACATAATCAATGATTCTAAATTTTGTAATTCAAATAGTAATG**

**Lminor_018706 ------------------------------------------------------------**

**Sp17g00070 GCAGAAACTAACGACCATTAATAAGTGATGATATATGCTTATGTGTTTTTAAACTCATGA**

**Lminor_018706 -----------------------GGTGACGA-----------------------------**

****** ****

**Sp17g00070 TTTTACAACTATGAACTAACACTAGATTGCAACTATTTGGCTAAATCAAACTATAAATTT**

**Lminor_018706 ------------------------------------------------------------**

**Sp17g00070 AGAAGCAAGTGTAAATAGGTTTAGGTCTATCCTAATGTAAGCTAATGTCTATTAATTAAT**

**Lminor_018706 ------------------------------------------------------------**

**Sp17g00070 TGATGTACTAGAGAAGGTACAGAAAGGAATAGGCTAAGATGATCAAACTAATAAATTGTG**

**Lminor_018706 -----------AGAAG--------------------------------------------**

*********

**Sp17g00070 TCTCTAATACTGATCATCTTAATCGTCTATGGTGAATCAGCCCTCTTCAGTTATAATGCT**

**Lminor_018706 ------------------------------------------------------------**

**Sp17g00070 CTTCAACAATTATCTTGAAATATTTGTATCTTATCTAAAAAAGAGAAGAGGGTCAATTGA**

**Lminor_018706 ------------------------------------AAGAAGAAGAAGAAG---------**

**** ** ****** ***

**Sp17g00070 GCCCTCCCCCTAACCCTCTTGATCTCTTTGATGCCTCATCAAACATAACATGATCACAAT**

**Lminor_018706 ------------------------------------------------------------**

**Sp17g00070 TAAAGTAGTAGCACAAAGTTTCTATGATCCACTAAGATCACGATAACAATCTTTAAAATT**

**Lminor_018706 --AAGAAGCAGAAGAAGCTTCC--------------------------------------**

***** ** ** * ** ** ***

**Sp17g00070 TTGCAATTCTATTTATCAATAAATAACTTTTAAGAGCTACTTAGTAACCAAAATGGGTTT**

**Lminor_018706 ------------------------------------------------------------**

**Sp17g00070 TGATGCATAAAACATAGCTTTCTTAAGTTTTTAAGTTTACTATAGATATCAATAGTGGTT**

**Lminor_018706 ------------------------------------------------------------**

**Sp17g00070 GATAGAATCCATGTCAATCATTGTTGTTAGTGGGCAATCTAACTTCAAAGCCAATTAATT**

**Lminor_018706 ------------------------------------------------------------**

**Sp17g00070 ACAACATGAAGGCATGAATATATCTCATCATAATTTTACTTGTCCATCTTACTAGTGGCT**

**Lminor_018706 ------------------------------------------------------------**

**Sp17g00070 AATTTTACAGACTCACTCGTATATAGAAACCATTAGTTCCAATTTTTTATGCACTAGATC**

**Lminor_018706 ------------------------------------------------------------**

**Sp17g00070 ATAAAGTTATACAAAAAATATTGGCAAAGGAAGTCATACTATCAATGTGTTTTAAGATCA**

**Lminor_018706 ------------------------------------------------------------**

**Sp17g00070 TATGCTGATTTCTTTGGTTTCCAGGGAATAATTTATGAAAAAATTAAATAAAAGGGCTTC**

**Lminor_018706 ------------------------------------------------------------**

**Sp17g00070 ATTTAATTATGAATTTGAGTGGATACTTAAAATCTCATGGCCCAATTAAAACATGCCGTG**

**Lminor_018706 ------------------------------------------------------------**

**Sp17g00070 AACATTCTTATAGACATATCTGGGGGCATTTAAAAAAATGCAGAATTTATATAAAGGCCT**

**Lminor_018706 ------------------------------------------------------------**

**Sp17g00070 TACTGAAATACGTTAATCTGAATGTTTTCGTACAAAGGCCCAGAATATATATAAAGCTCT**

**Lminor_018706 ------------------------------------------------------------**

**Sp17g00070 GCATTTGGGGATAGGGACGGATCCGGCCGCGCGATCCACCCAAGGATATAAGGCTGGGGT**

**Lminor_018706 ------------------------------------------------------------**

**Sp17g00070 GGGCACCCATGTCGACACGTGGACGAACCGGGTACACGGCATGGACACGTGGCTGACTGA**

**Lminor_018706 ------------------------------------------------------------**

**Sp17g00070 CATAGGGACGACTCTCGACACGTGAGAGAGGCCGTACCGGGGGGAGCTGGAGTGGGTGGC**

**Lminor_018706 ------------------------------------------------------------**

**Sp17g00070 GGATCCTGCCGCGGCAGGTCCCGCACGCGGCTCCGCGGCCGAGGCGGCGATTGATGGCTC**

**Lminor_018706 --------------------------------------CCGAGACGACGA----------**

******* ** *****

**Sp17g00070 AATGAAAAGCCTGCCGCCTGCGCCTCCAGTCCGATGGCGAAG-AAGTGAAGAGAGAGAGA**

**Lminor_018706 -------------------------------CGAAGAAGAAGAAAACGAAGAAGAAGATG**

***** * **** ** ***** *****

**Sp17g00070 GAGAGAGAGAGAGAGAGAGAGAACCAGTAGAGCGAGAGAGTGAAGGAGAGATGGAGACCA**

**Lminor_018706 AGGAGGAAGAGGAAGAGAGAGAAGAAGAAGAG---------GAGGAGGAGAAGGGTTTTA**

***** **** ********** ** **** ** * **** ** ***

**Sp17g00070 GTAGAGAGAGAGAGAGAGAGAGAGAACCAGTAGAGCG----AGAGAGTGAAGGAGAGATA**

**Lminor_018706 GT-------GAGAGACAGAGAGAGCACCCGTTCATCGTCACAGAGCCTGGGGA-------**



**** ****** ******** *** ** * ** **** ** ***

**Sp17g00070 GAGACCAGTAGAGAGAGAGAGAAAGAGAGATGTCTGACTCCCCGCCGTCGCAGACCCCAC**

**Lminor_018706 ------AGTAGCGAGAGGGAAGAAGAACG--GGCTGGA----------------------**

******* ***** ** **** * * *****

**Sp17g00070 CAGCCTTTTCCCTCCTCCTCCTCTGCATCCACCACCGCTGAAGGGAGGCGGTGGTGGCGC**

**Lminor_018706 -------TTATCTTTTCCACCTCTACG---------------------------------**

**** ** *** ***** ***

**Sp17g00070 CGGAGAACCGACCCATCGCCGCCGGCAGCTTCCCCGGCA---GCCCACCTCGCCGGAATT**

**Lminor_018706 --------------ATCTCTGCCGAGAGTTTCTCTTGCAGGTGCAAGCCATGGCGA----**

***** * **** ** *** * *** ** ** * ****

**Sp17g00070 GAGGCCGAGGCCTATCACAGCGGCGGCGGCGGCACCTGCGAGTTCCCAAAACGAGAGGGA**

**Lminor_018706 GAGACCGAGGCC-------------------------ACAAGTGTCCAACA---------**

***** ******** * *** **** ***

**Sp17g00070 GAGAGGGAGAGGGAGGGCTGCATCTCCGTCTCTCTCT-ACATGCCTCTGGTTCGC--TCT**

**Lminor_018706 ---------AAGGTGGAACCTTTCTCTCTCTATCTCTAACCCGCCTC--GTTAGCCGTTT**

*** ** ** **** *** ***** ** ***** *** ** * ***

**Sp17g00070 CTATCTTTGTCCGTGTTGTGTTCCTCGTTGTAAGAGCTCTCCTTCCTCCTGCTGCCAGCA**

**Lminor_018706 CTCTCTCTATCCTTCATGGG---CTTGATGTG-----TCTCTTTCCTTC-----------**

**** *** * *** * ** * ** * *** **** ***** ***

**Sp17g00070 GGAAGGAGTCAATTAATCGCATCGGAGAAAGGAAGCTGACAGGCGGGCGGGTTCTCGTAG**

**Lminor_018706 ------------------------------------------------------------**

**Sp17g00070 AAGTCTCTCCCTCAGGTGTTGGGCGGAGGACGGTGTGACGTGATTTGATCGATTACAGCG**

**Lminor_018706 ---TCTATCTCTAAA---------------------------------------------**

***** ** ** ***

**Sp17g00070 GCTTTTGCCCCCCCCGCCTCTTCTTCTTCCACTGCCAATCTGTGCTCTTTCGCAAGGGTT**

**Lminor_018706 --------CCCCCTCGTTTTTCTCTCTATCCTT-------------------CAAGGGTT**

******* ** * * *** * * **********

**Sp17g00070 TCACTGTTAGTCTACCCCCCCCTTCTCTCTCTCTCTCTCTCTACTGGTTTCTCCCTCTTC**

**Lminor_018706 TGA--------------------TGTCTTTCTTTCTCTCTCTA----------CCTC---**

*** * * *** *** ********** ******

**Sp17g00070 ATGGTCTCTCTC-TCTCTCTCGACTGGTTTCTCCTCTTCATAGGTCTCTCTCTCTCTCTC**

**Lminor_018706 -TGACCTACCTCGTTTCTCTC--------TCTGCGTTTCATGGGTTTGTTTTTGTCTTTC**

**** ** *** * ****** *** * ***** *** * * * * *** ****

**Sp17g00070 TCTCTCTCTCTCTCTCTTTCTCTCTCTACTGGTTTCTTGGCTCTCTATCTATCCCTGCAT**

**Lminor_018706 TTTCTCTCCATCTCTAACCAAC------CTAGTTT-----CTCTCTATC-----------**

*** ****** ***** * ** **** ***********

**Sp17g00070 GACAGCGCATGGAGCAGAGTGAGAGTTCACATCGCCGCTAACTCTGCCCCCCCCCCCCTC**

**Lminor_018706 ----GTTCATGGGTTCGA----------------------------------------TG**

*** ***** ** ***

**Sp17g00070 TCTCTCTCTCTCTCTCTCGCTTTTCCTCACAATCTATGGATGTGAGAGAAGGACCGTCAA**

**Lminor_018706 TCTTTCTTTCTCTCTCTATCCTT-----------CATTGATTTGA---------------**

***** *** ********* * ** ** *** *****

**Sp17g00070 CGCTCATGAAATAAAGACGGCCGAAGGGATTCCGACGAGCAACCACTACAAGTTTGGTGC**

**Lminor_018706 ------------------------------------------------------------**

**Sp17g00070 AGAGGAGCCCCTCAACTTCTCCAGTTCAAATAAATATCACAGAACATCTGAATAGTTCAA**

**Lminor_018706 -----CGTCTTTCTTCCTCTCTACCTC---------------------------------**

*** * ** * **** * ****

**Sp17g00070 GCATCAGATAAAGTTTATACAAAATGGGCATGCTCCAATTCCATCGAAGATCCAGAAAGT**

**Lminor_018706 ------------------------------------------------------------**

**Sp17g00070 CGTGTTCTTGGGGATAAGTTTGGTGTTTTTTAGTTAGATTTCGCTCTGAATTATGAGTAA**

**Lminor_018706 --------------------------TTATCCGCCATGTTTCTCTCT-------------**

**** * * * **** ******

**Sp17g00070 TATAATGGGTATATTTTATGTAGATTATGACACCGATGTGTTCACATGTCCCCTCGGAAG**

**Lminor_018706 ------------------------------------------------------------**

**Sp17g00070 CACTATCGTTTTAAGGTTGTCTTCTGGAACGGGTGGCGGGGAGGGGTGGGCGGCAGGGGC**

**Lminor_018706 --CTATCTCTT-------------------------------------------------**

******* ****

**Sp17g00070 CGCCGGCCGAGGCGGCGGTGGGTCCAGCGCCGTCGCCGCTTAGTCACCCATCACAGGTGA**

**Lminor_018706 ---------------------------------------------ACCCATT--------**

**********

**Sp17g00070 TCTCGAGCAAGCCAGAGTTGCAGACATGCTCAGGAGGGGCAATCTGGAGATTTCTAGCTC**

**Lminor_018706 -----------------------------------------------TGGTTTCTATCTC**

*** ****** *****

**Sp17g00070 CCCGTCCGCAGTACTTAAATGCAGCTTCAGCGGCGGTGAGATCGCCGGAGAAGCGCCGGA**

**Lminor_018706 C-----------------------------------------------------------**

*****

**Sp17g00070 GGAAGGAGTTGAAGAAGCCGAAGAAGAAGAAGAAGAAGAGTAAACCCTAGAAGAAGAGGA**

**Lminor_018706 ------------------------------------------------------------**

**Sp17g00070 AGATGGATCCCGCCGACGAGTTTTCAGCGGCGGCGAACCCGTTCAGATGCTGGGACCTGA**

**Lminor_018706 ------ATCC----------TTCTC-----------------------------------**

****** ** ****

**Sp17g00070 GGATGGGGCACCCGAACAGGCTGATCGAGGCGGCGGCGTCGCTGCTAGCCGCCCAGGTGG**

**Lminor_018706 ------------------------------------------------------------**

**Sp17g00070 GCAGGCGCCGCCGGGGAGGGGGGGAGCTGGAGGAGATGTTCCAGGCGTACGGAGTGAGGT**

**Lminor_018706 ------------------------------------------------------------**

**Sp17g00070 ACCAGACGGTGGAGAAGATCGGGGAGCTGGGGTTTACGGTTAGCACGCTGGCGGCGATGA**

**Lminor_018706 ---------------------GGAATCATGGGTTT-------------------------**

**** * * ********

**Sp17g00070 GGGAGGAGGAGCTGGAGGACATGATGGCCGCCCTGTGCCATGTCTTCCGGTGGGAGCTCC**

**Lminor_018706 ------------------------------------------------------------**

**Sp17g00070 TCGTCGGAGAGAGGTACGGCATCAAAGCCGCCGTCCGCACCGAGCGGCGGCGGCGGCAGC**

**Lminor_018706 ------------------------------------------------------------**

**Sp17g00070 TCGACGGGTTCTTCCAACTGGACGAGGAGGAGCAGCGGCGCCGCCGCCATGTCGTCTCCC**

**Lminor_018706 ------GATTCTTTC-------------------------------------CTTCTCTC**

*** ***** * * **** ***

**Sp17g00070 CCGACTCCGGCGCCCTCGACGCCCTCTCCCAGGAAGGTGAGCACCCACCTGCTCTCCGTC**

**Lminor_018706 TCTAC----------------CTCTAACCCAGTAAGGCAGTAACCCA------------C**

*** ** * ** ***** **** ***** ***



**Sp17g00070 CTCTTCCATCCCCGCCGGCTCTTCATTCCTGG--TTCATTCGCAGGCCTGTCGGAGGAGC**

**Lminor_018706 CTCGTTTATCTCT-----CTATTTTTTCATGGGTTTGATTTG------------------**

***** * *** * ** ** *** *** ** *** ***

**Sp17g00070 GGGTCCAGCAGGAGAGGGAGGCGGTGGCCAGCGGCGGCGAGGCCTGGGAGGGCCACAAGA**

**Lminor_018706 ------------------------------------------------------------**

**Sp17g00070 AGAAGAAGACGAACACCAGCACCAGGAGGAACAAGAAGACGACGACGAAGAAGAAGAAGA**

**Lminor_018706 ------------------------------------------------------------**

**Sp17g00070 ACGAGGAGGAGGAGGAAGACGGCGAGGAGGAGGACGACGACGACGATGAGGAAGAAGACG**

**Lminor_018706 ------------------------------------------------------------**

**Sp17g00070 GTGAAGGAGAGTGCGAGGGGAAGGTCGCCGGCTGCGGCGCCGCCGCCGGCGGGGAGAGGC**

**Lminor_018706 ---------------------ACATCTCTGTCT---------------------------**

*** ** * * ****

**Sp17g00070 AGCGGGAGCACCCGTTCATCGTGACGGAGCCCGGAGAGGTTGCCCGGGGAAAGAAGAACG**

**Lminor_018706 ------------------------------------------------------------**

**Sp17g00070 GCCTCGACTACCTCT-TCCACCTCTACGACCAGTGCCGCGACTTCCTCCTCCAAGTCCAG**

**Lminor_018706 -------CTGTCTCTCTCTACCTGTA-------------GTCTATCTC-----------G**

**** **** ** **** ** * ** *** ***

**Sp17g00070 TCCATCTCCCGCGAACGCGGCCACAAGTGCCCCACCAAGGTCCAGAACCCTACTCTCTCT**

**Lminor_018706 TTTATCTCTC------------------TATCCGTCATGGTTTGATGTCATTCTTTCTCT**

*** ***** * ** ** *** * * ** *******

**Sp17g00070 CTCTCTCTCTCTCTCTCTCGCTCTCGCTCTACTGAGATGCGGAGGGGCTGGCGGCGCCGC**

**Lminor_018706 CTCTACGTTTCTT-------------------------------------GCGGC-----**

****** * *** *******



**Sp17g00070 CGTGCAGGTGACGAACCAGGTGTTCAGGTACGCGAAGGAAGCGGGGGCGAGCTACATAAA**

**Lminor_018706 ----CAGGTGACGAACCAGGTGTTTAGGTTTGCAAAGGAGGCGGGGGCAAGCTACATAAA**

********************** **** ** ***** ******** *************

**qLFY-F**

**qLFY-R**

**Sp17g00070 CAAGCCGAAGATGAGGCACTACGTGCACTGCTACGCGCTGCACTGTCTGGACGAGGAGGC**

**Lminor_018706 CAAGCCGAAGATGAGGCATTACGTGCACTGCTACGCGCTGCACTGTCTGGACGAGGAAGC**

******************** ************************************** ****

**Sp17g00070 GTCGAATGCACTGAGAAGGGCGTTCAAGGAGAAGGGGGAGAACGTCGGAGCGTGGCGGCA**

**Lminor_018706 GTCGAATTTGCTGAGAAGGGTCTTCAAGGAGAAGGGAGAGAATGTGGGGGCGTGGAGGCA**

********* ********** ************** ***** ** ** ****** ******

**Sp17g00070 GGCCTGCTATCAGCCACTGGTGGCGCTATCAGCGCGCCACCATGGATGGGACGTGGACGC**

**Lminor_018706 GGCGTGCTATCAGCCGCTCGTGGCTATGGCGGGGAGAAGGCATGGATGGGATGTGGACGC**

***** *********** ** ***** * * * * * *********** **********

**Sp17g00070 ACTCTTCAACTCACACCCGCGGCTCTGCATCTGGTACGTGCCCACCAAGCTCCGCCAGCT**

**Lminor_018706 CATGTTCAATTCGCAGCCAAAGCTCGCGATTTGGTACGTGCCCACGAAGCTCCGGCAGCT**

*** ***** ** ** ** **** ** ************** ******** *******

**Sp17g00070 CTGCCACCTCGCCCGCAGCGCCGCCGTTGCCACAGCGCCGCCGGCCCA---------CGG**

**Lminor_018706 CTGCCACTTGGCTCGCAGCGGCGCCGTCAGTGCCGCGCCGCCGGCCAACTGCTTGAGCGG**

********* * ** ******* ****** * ************ * *****

**\**

**Sp17g00070 CGGCATGCCTTACCTCTTCTAG**

**Lminor_018706 CGGCGTAGCGTACATCTTCTAG**

****** * * *** **********


Supplement: Supplementary file 1 [file plants-12-00872-s001.zip › Supplemental files for Acosta et al. Plants'23_final2/FileS3.docx]
