# Supplementary material for: Optimization of Molecular Methods for Detecting Duckweed-Associated Bacteria
Source: Plants (Basel). 2023 Feb 15;12(4):872. doi: 10.3390/plants12040872 (PMC9965182; doi:10.3390/plants12040872)
Supplement: Supplementary file 1 [file plants-12-00872-s001.zip › Supplemental files for Acosta et al. Plants'23_final2/FileS5.pptx]

## Slide 1
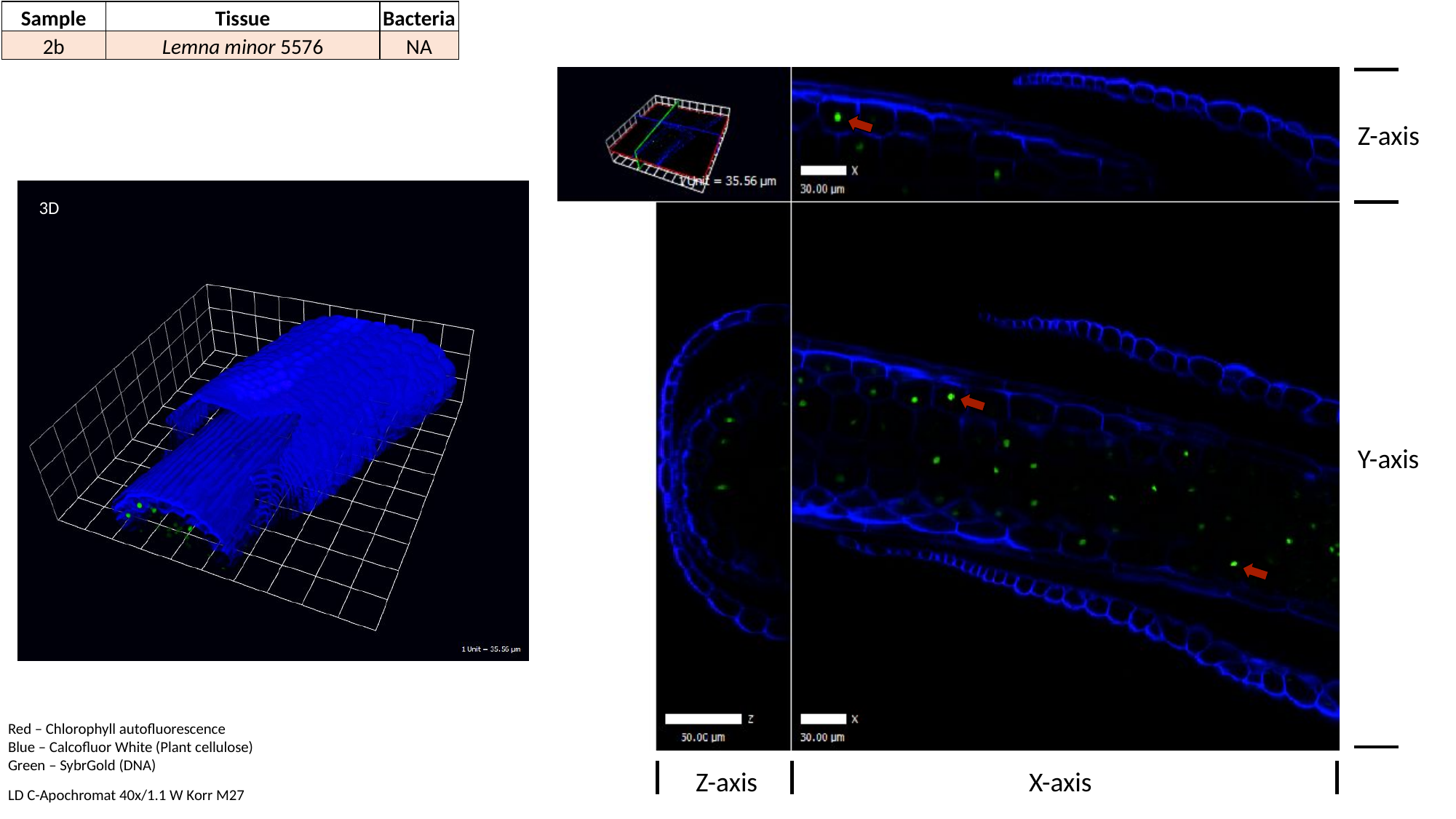

| Sample | Tissue | Bacteria |
| --- | --- | --- |
| 2b | Lemna minor 5576 | NA |
Z-axis
3D
3D
Y-axis
Red – Chlorophyll autofluorescence
Blue – Calcofluor White (Plant cellulose)
Green – SybrGold (DNA)
Z-axis
X-axis
LD C-Apochromat 40x/1.1 W Korr M27

## Slide 2
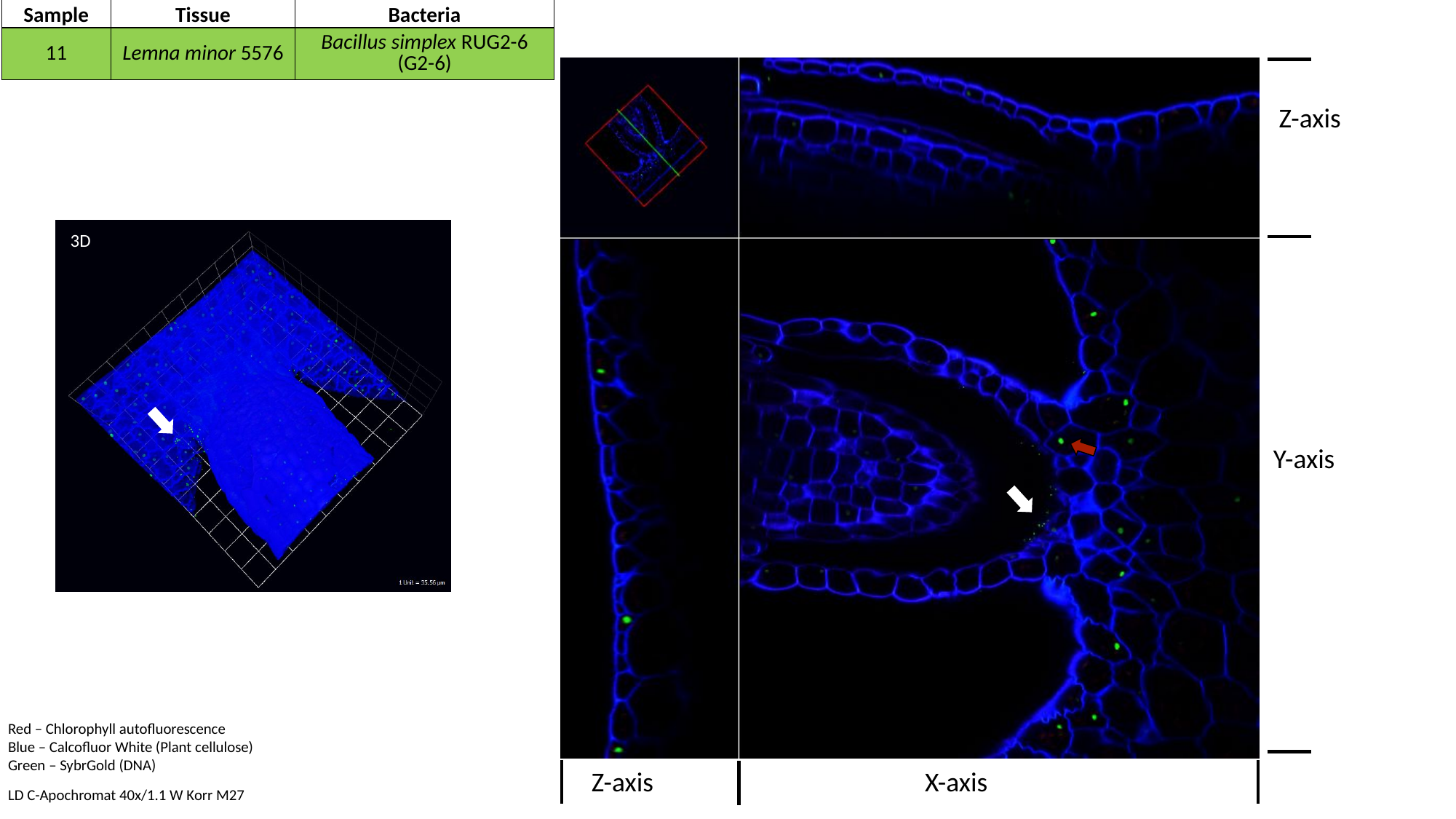

| Sample | Tissue | Bacteria |
| --- | --- | --- |
| 11 | Lemna minor 5576 | Bacillus simplex RUG2-6 (G2-6) |
Z-axis
3D
Y-axis
Red – Chlorophyll autofluorescence
Blue – Calcofluor White (Plant cellulose)
Green – SybrGold (DNA)
Z-axis
X-axis
LD C-Apochromat 40x/1.1 W Korr M27

## Slide 3
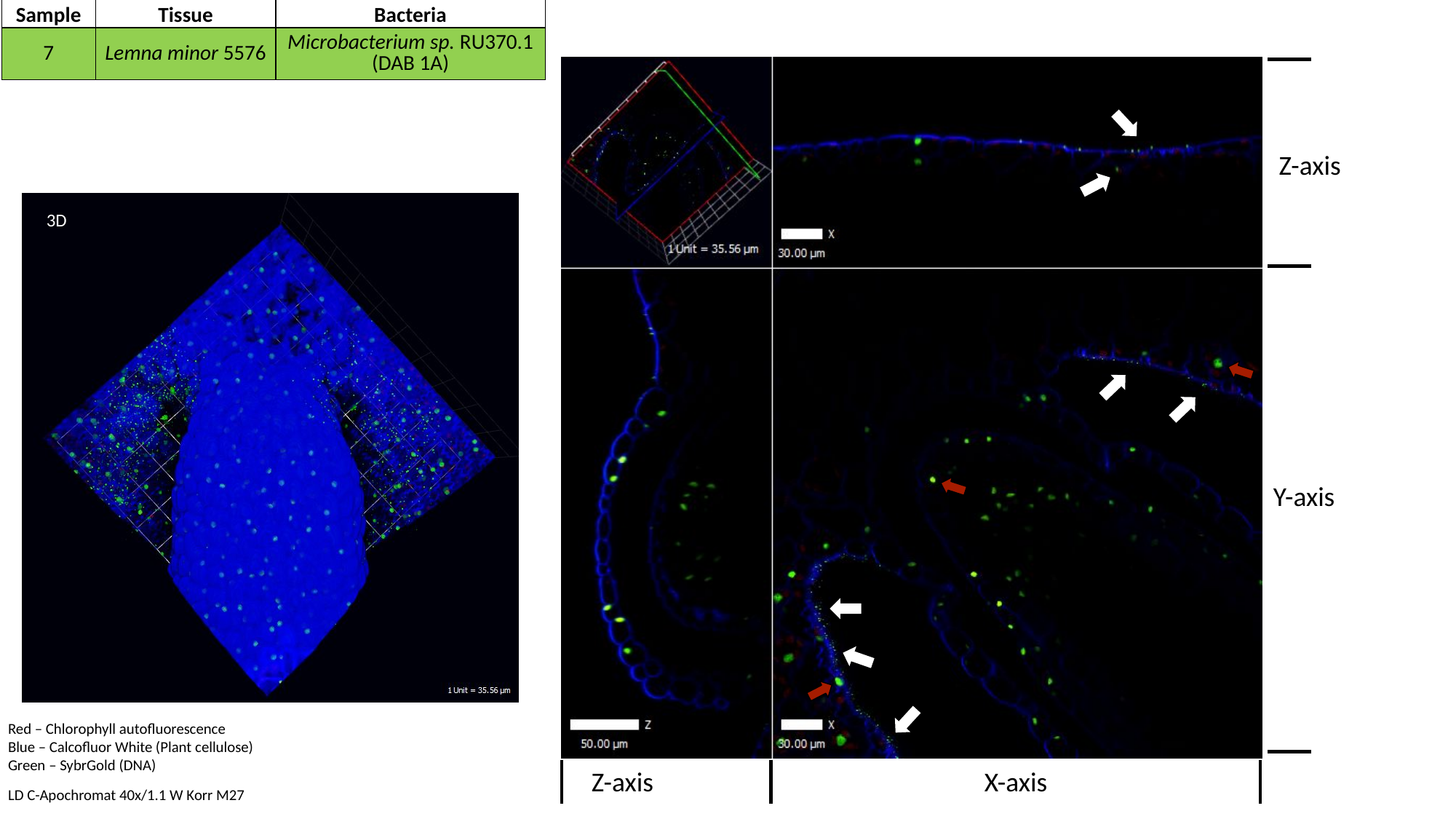

| Sample | Tissue | Bacteria |
| --- | --- | --- |
| 7 | Lemna minor 5576 | Microbacterium sp. RU370.1 (DAB 1A) |
Z-axis
3D
Y-axis
Red – Chlorophyll autofluorescence
Blue – Calcofluor White (Plant cellulose)
Green – SybrGold (DNA)
Z-axis
X-axis
LD C-Apochromat 40x/1.1 W Korr M27

## Slide 4
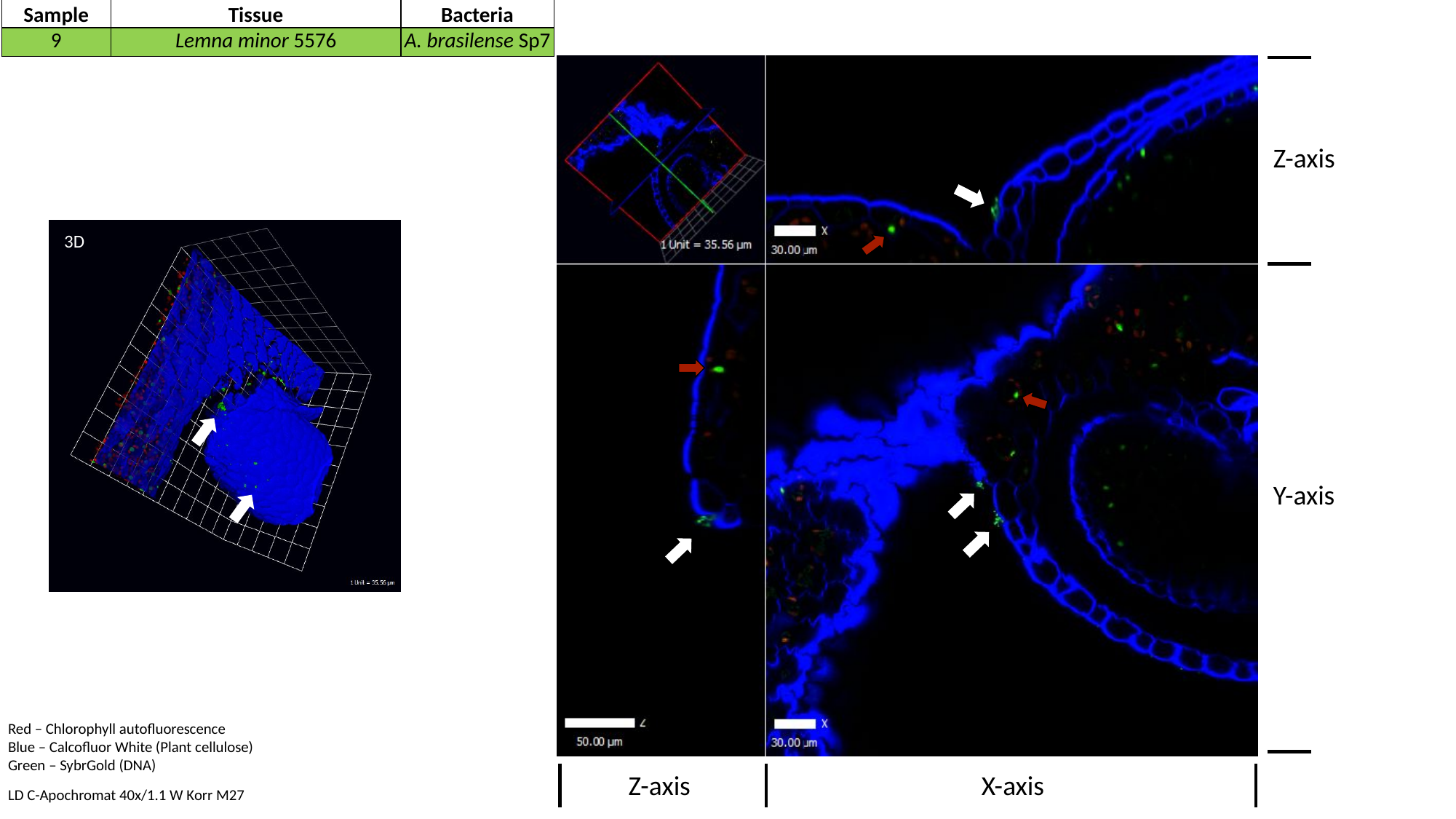

| Sample | Tissue | Bacteria |
| --- | --- | --- |
| 9 | Lemna minor 5576 | A. brasilense Sp7 |
Z-axis
3D
Y-axis
Red – Chlorophyll autofluorescence
Blue – Calcofluor White (Plant cellulose)
Green – SybrGold (DNA)
Z-axis
X-axis
LD C-Apochromat 40x/1.1 W Korr M27

## Slide 5
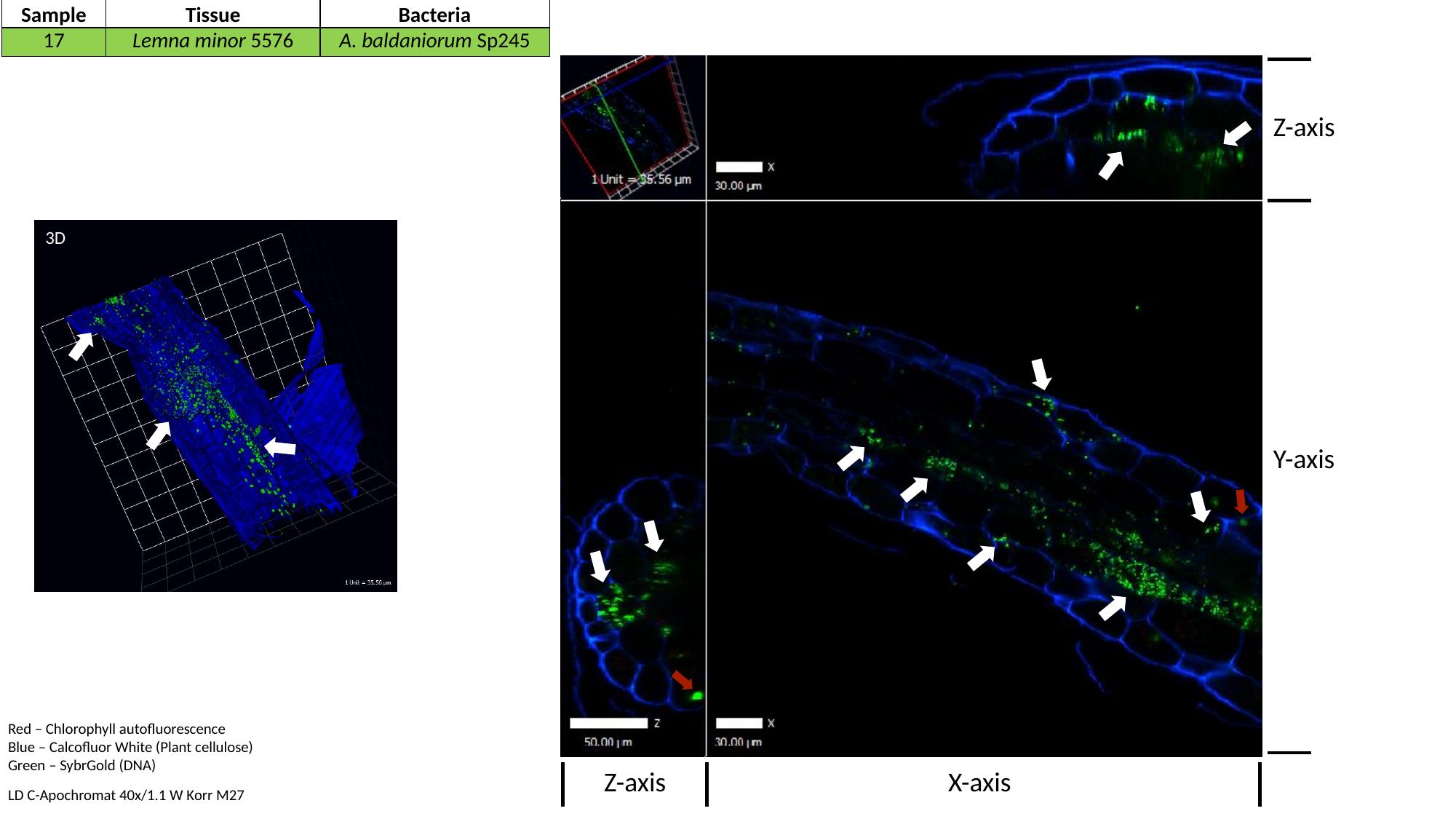

| Sample | Tissue | Bacteria |
| --- | --- | --- |
| 17 | Lemna minor 5576 | A. baldaniorum Sp245 |
Z-axis
3D
Y-axis
Red – Chlorophyll autofluorescence
Blue – Calcofluor White (Plant cellulose)
Green – SybrGold (DNA)
Z-axis
X-axis
LD C-Apochromat 40x/1.1 W Korr M27
