# Supplementary figures and images for: Optimization of Molecular Methods for Detecting Duckweed-Associated Bacteria
Source: Plants (Basel). 2023 Feb 15;12(4):872. doi: 10.3390/plants12040872 (PMC9965182; doi:10.3390/plants12040872)

**A**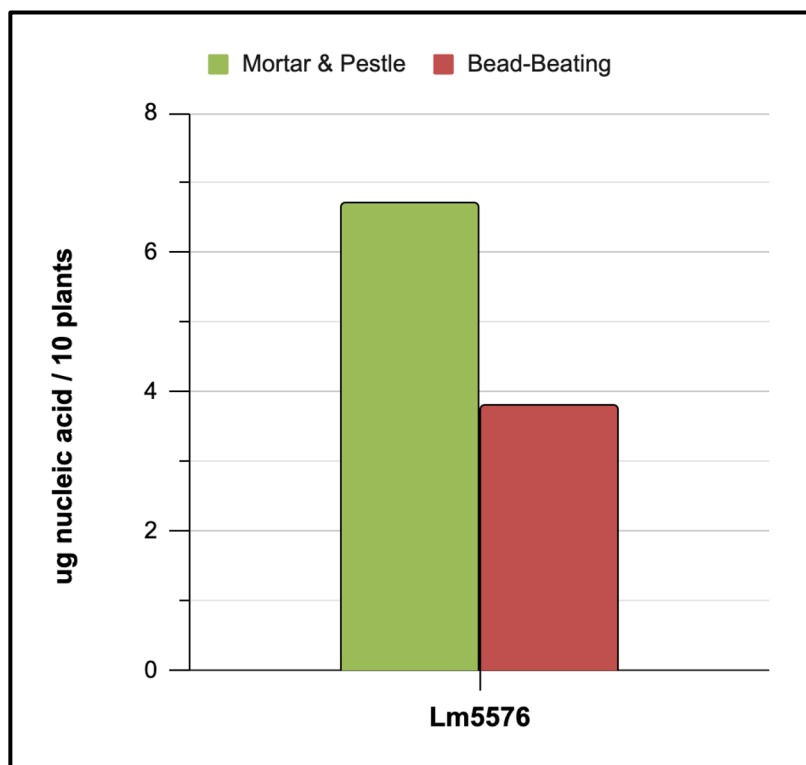**B**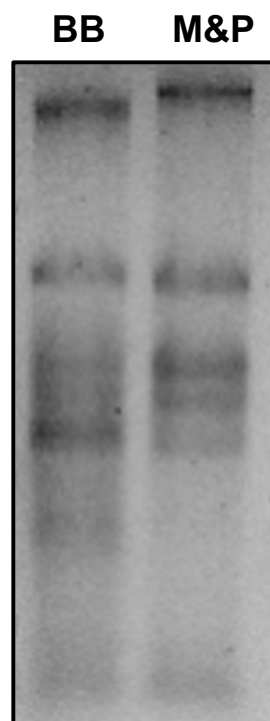**C**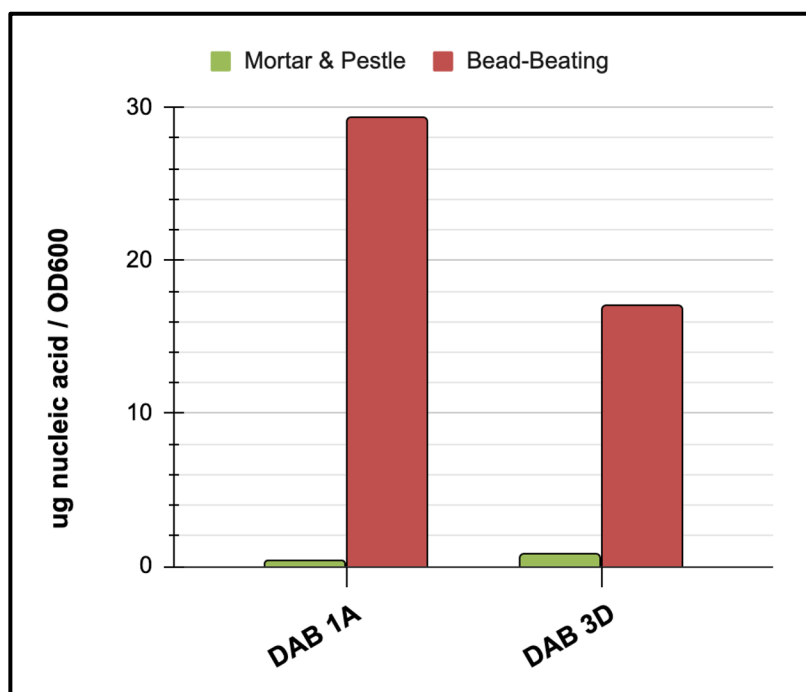**D**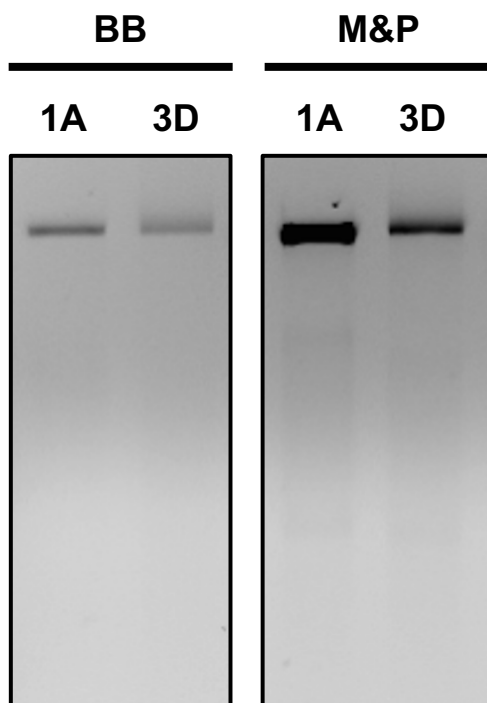**Figure S1**

**A**

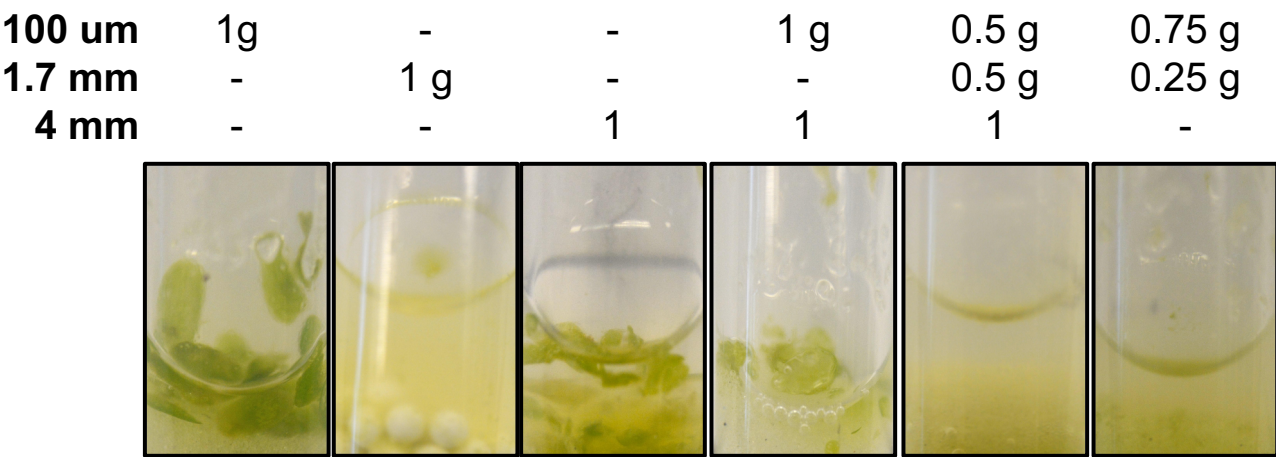

**B**

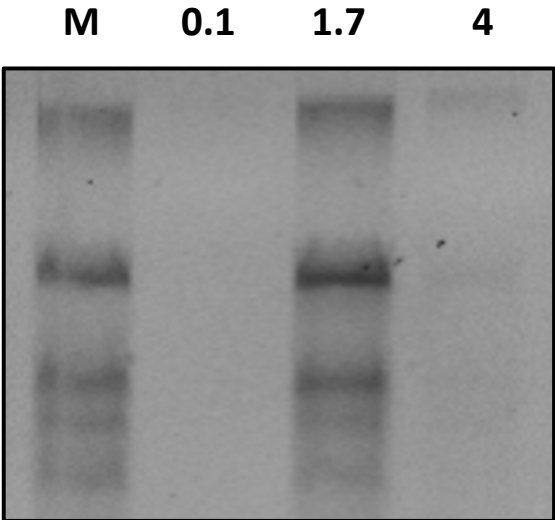

**C**

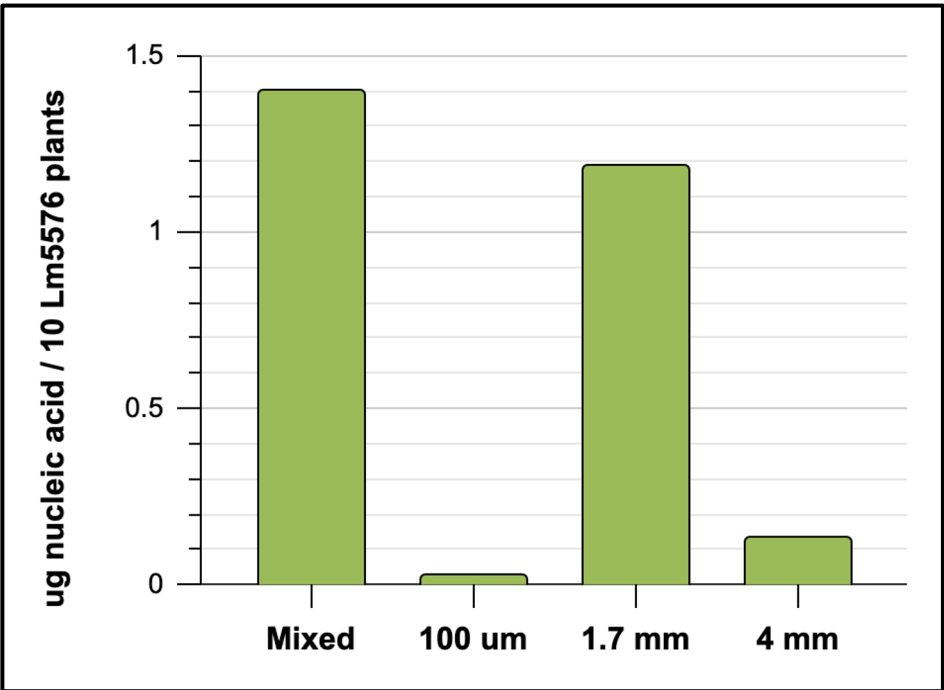

**Figure S2**

**A**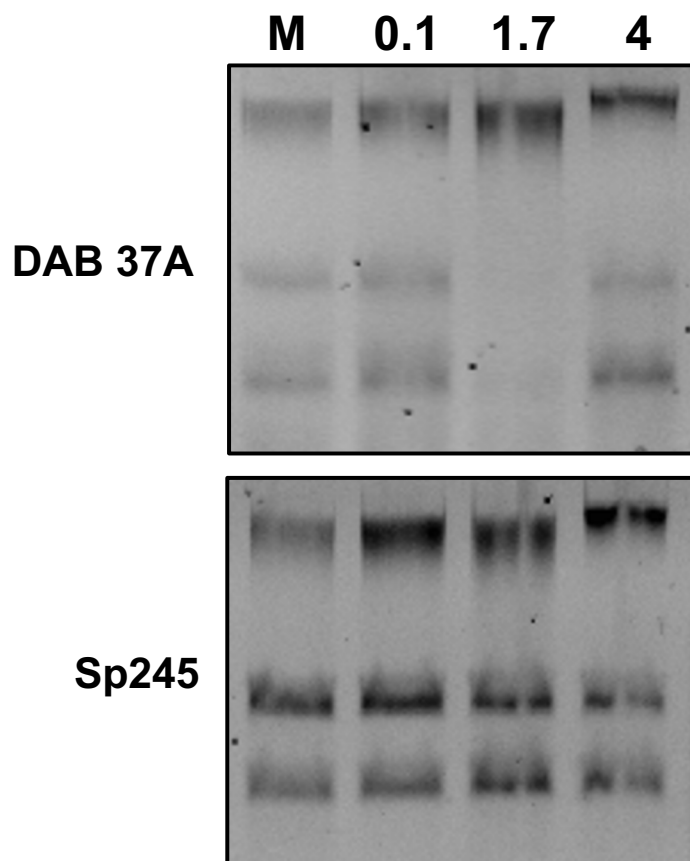**B**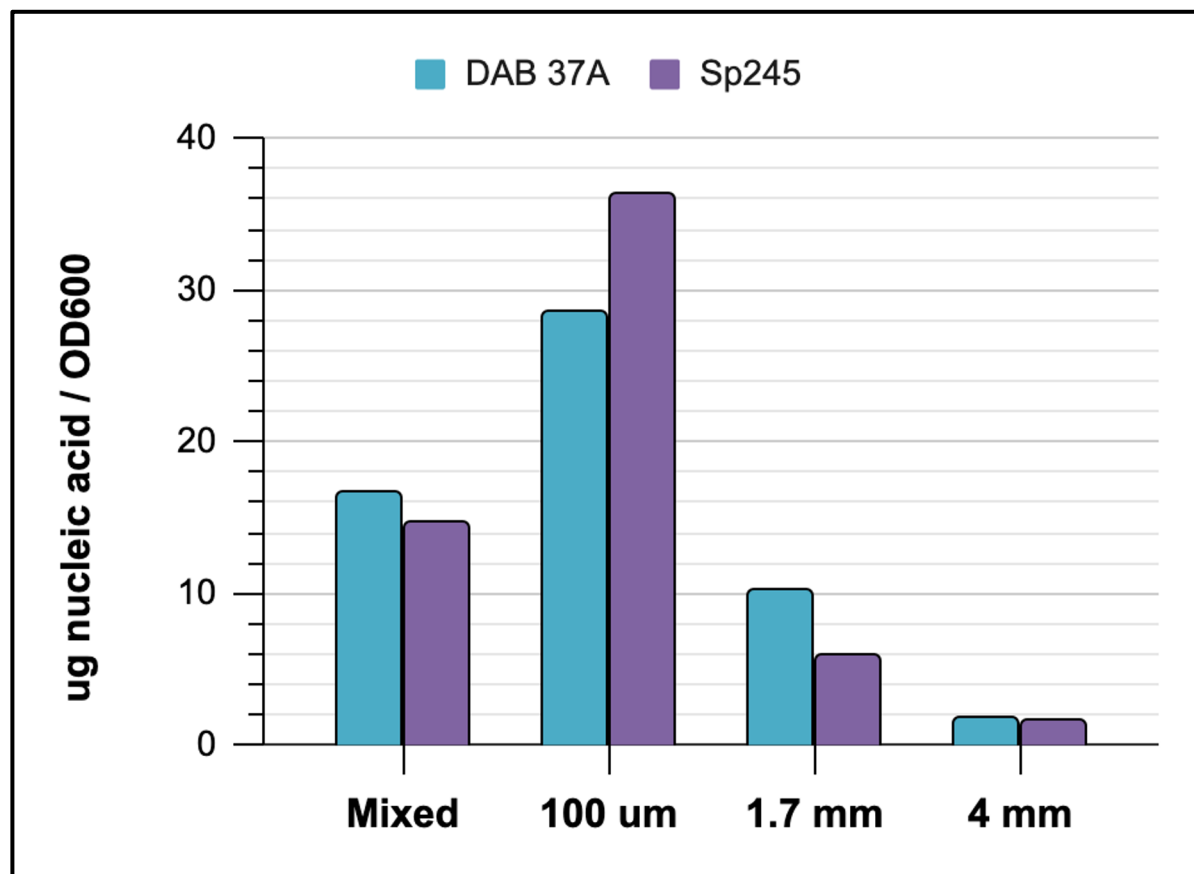**Figure S3**

**A**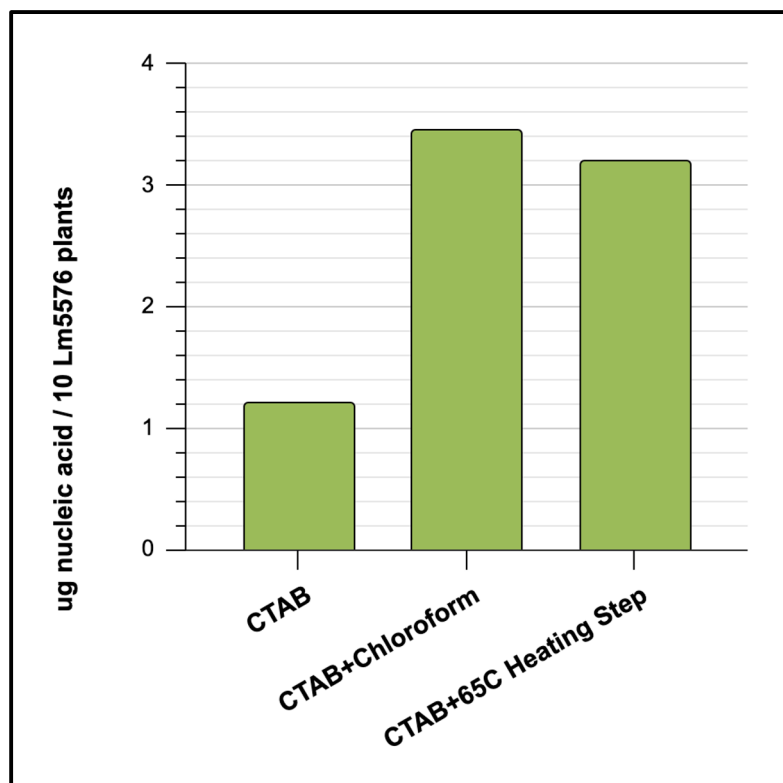**B**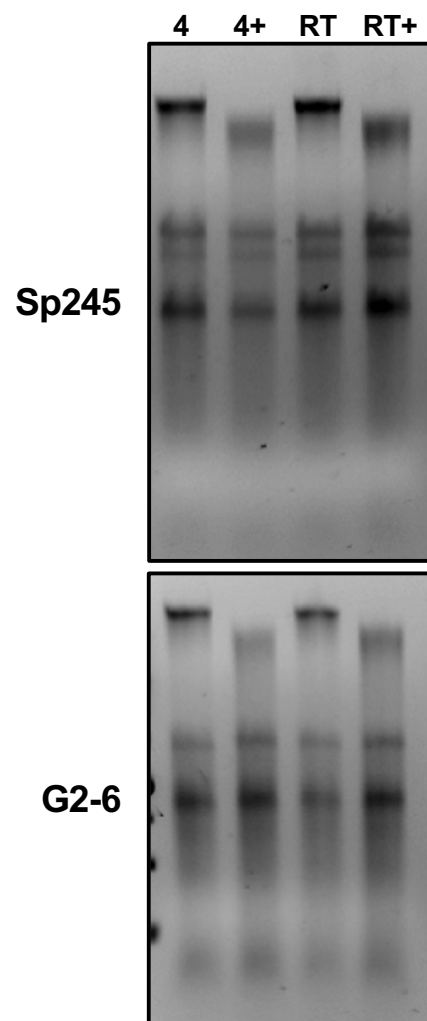**C**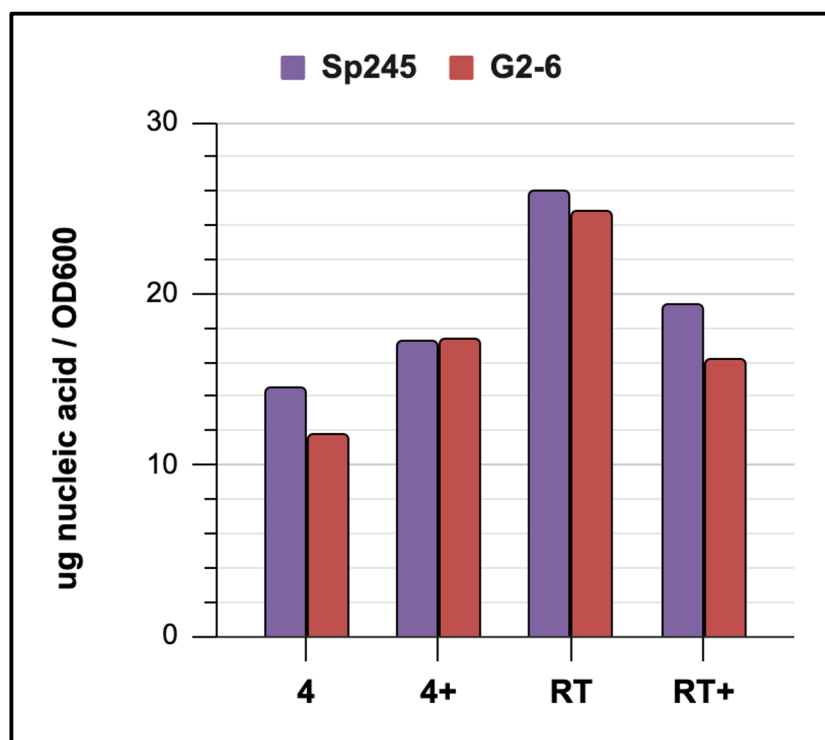**Figure S4**

**A** **B**

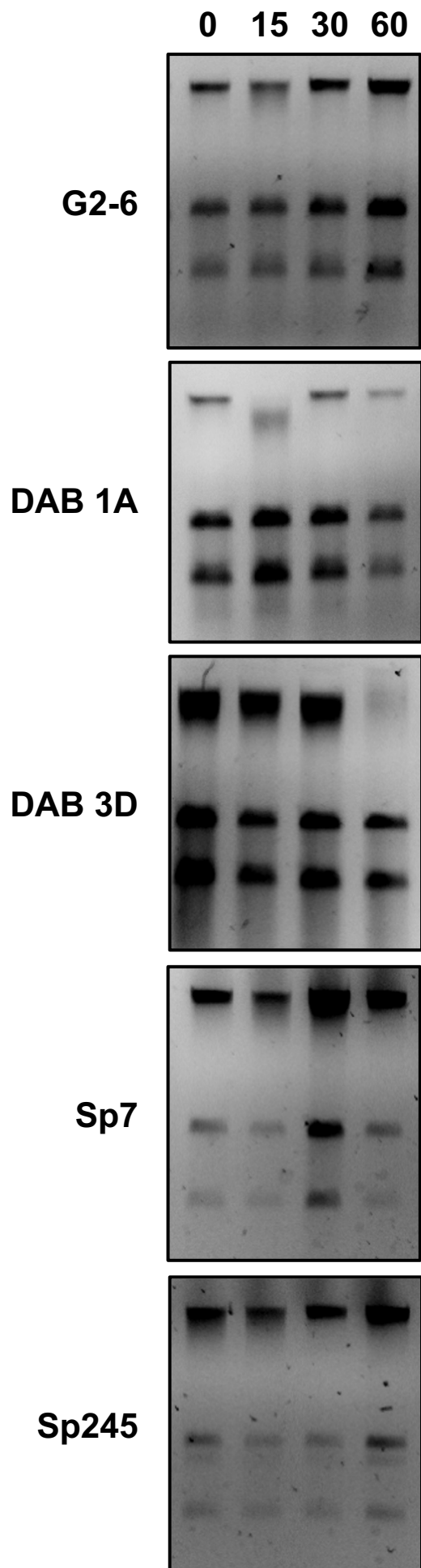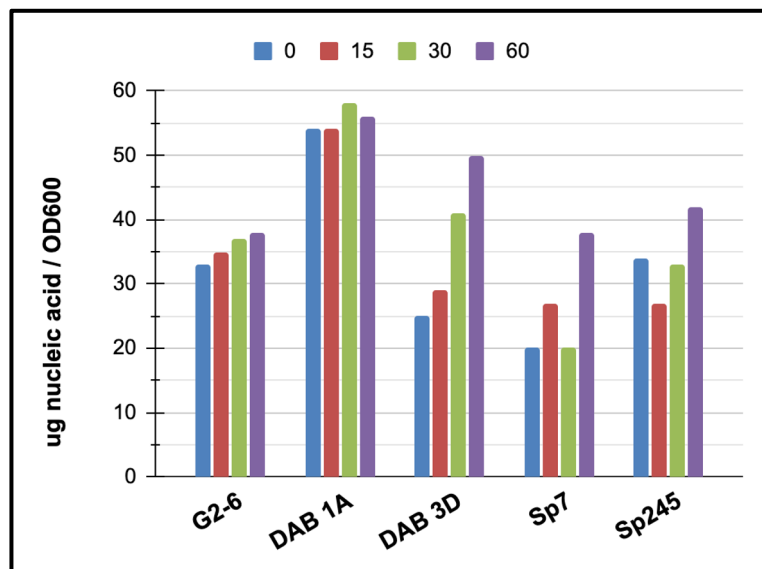

**Figure S5**

**A**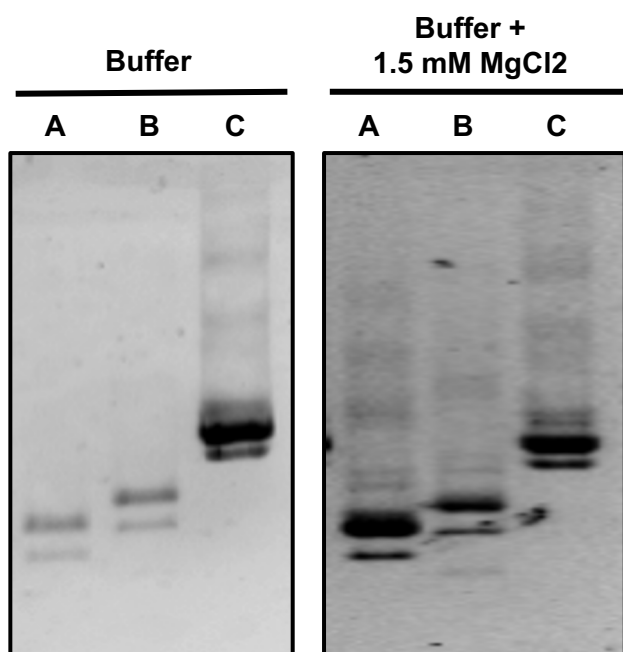**C**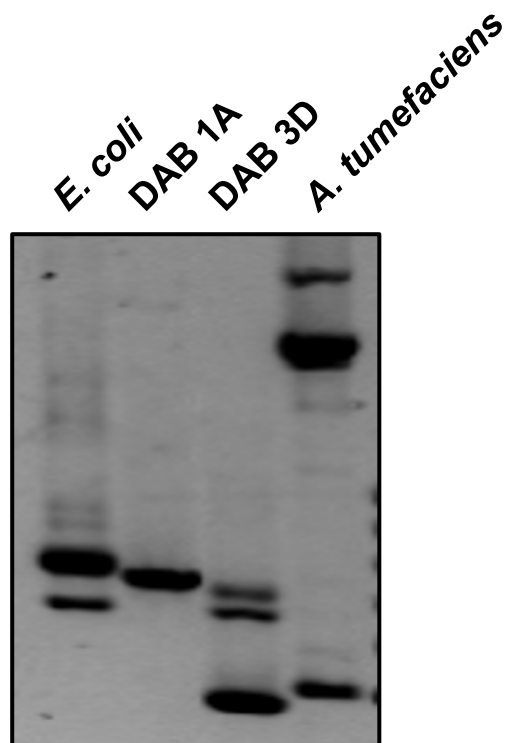**B**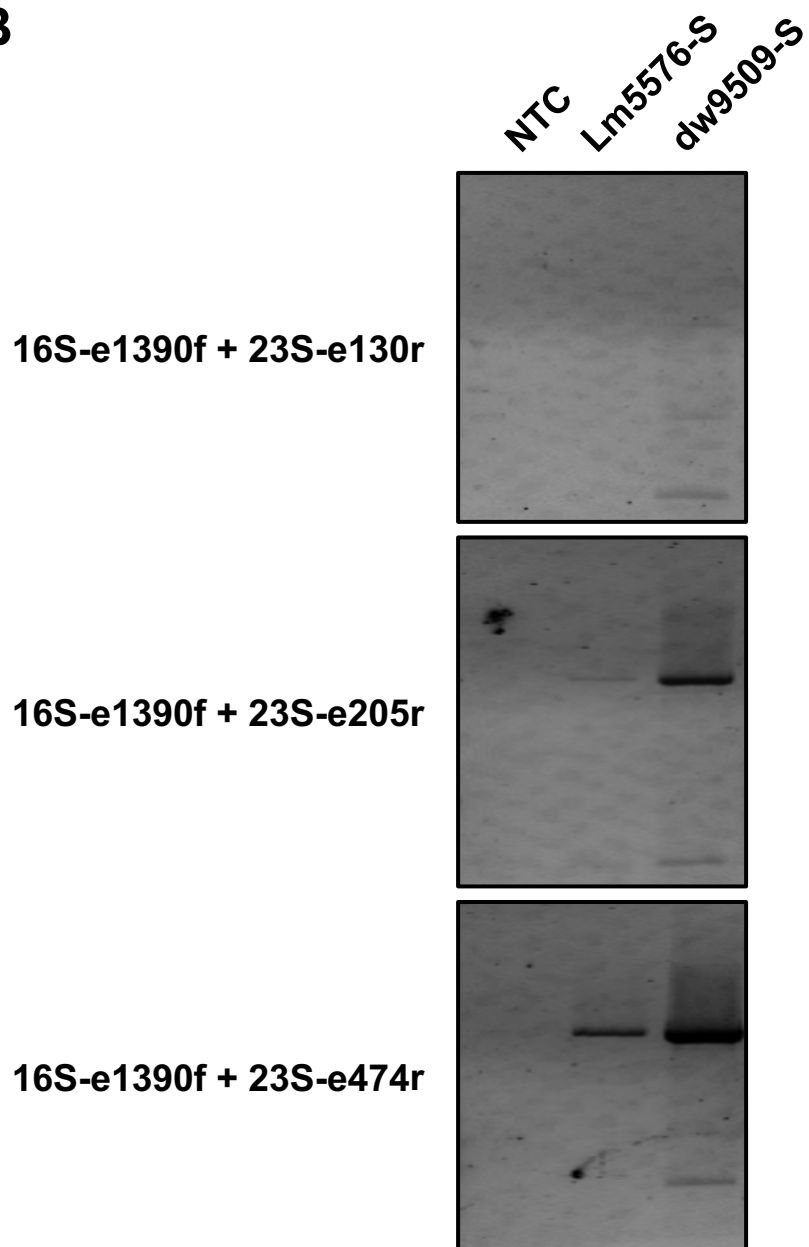**Figure S6**

**A**

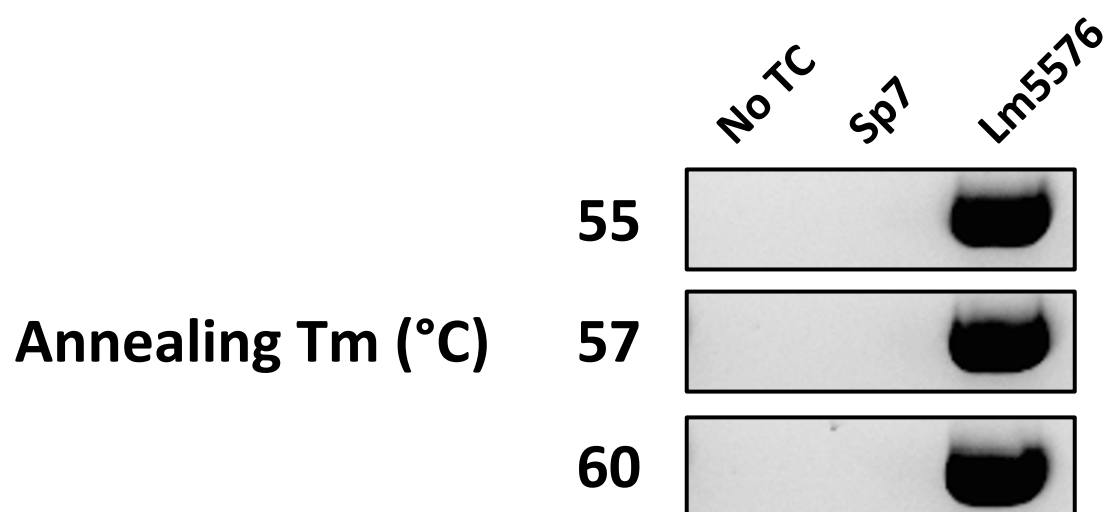

**B**

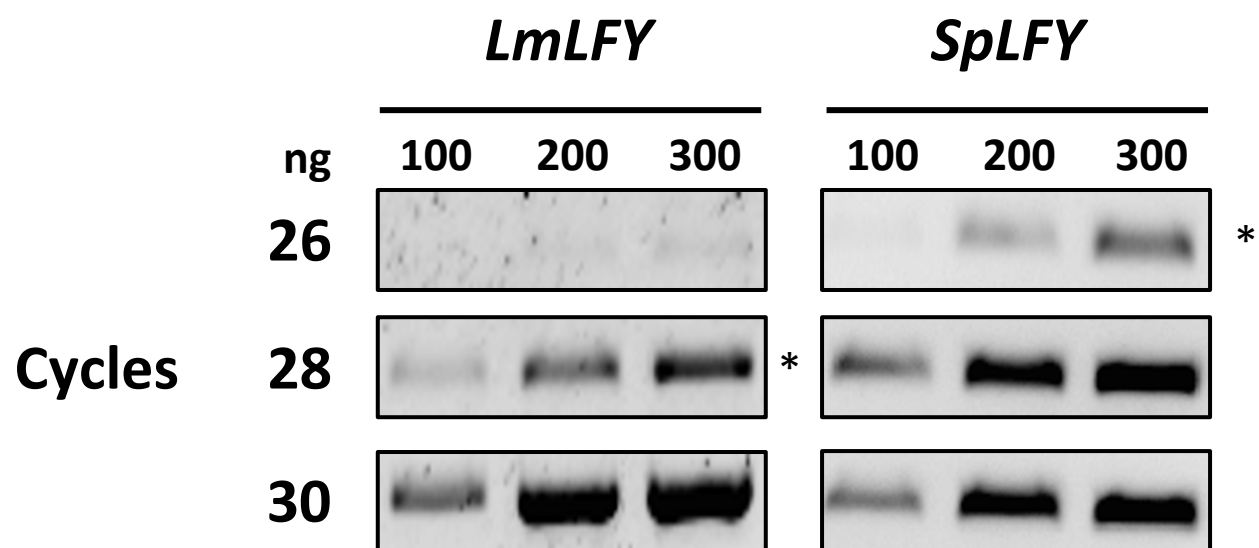

**Figure S7**

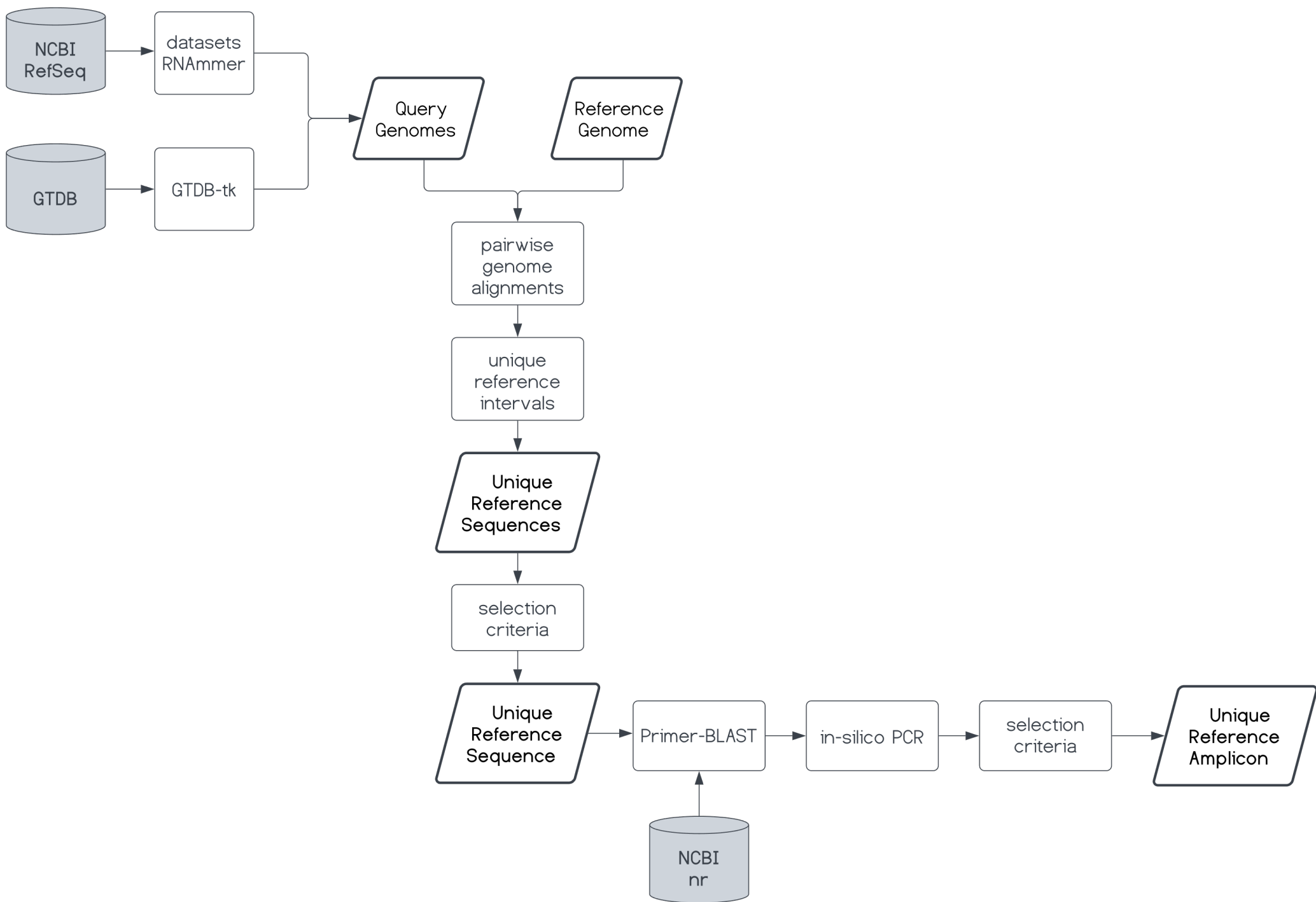

**Figure S8**

Supplement: Supplementary file 1 [file plants-12-00872-s001.zip › Supplemental files for Acosta et al. Plants'23_final2/Supplemental Figures 1-8_final.pdf]
